# Epidemiology, seroprevalence, and circulation of Chikungunya virus in Southern Africa (SADC region): A systematic review

**DOI:** 10.1371/journal.pntd.0014438

**Published:** 2026-06-16

**Authors:** Siphamandla Lamula, Ndivhuwo Ramatsitsi, Lisa Buwa-Komoreng

**Affiliations:** 1 Infectious Diseases and Medicinal Plants, Botany Department, Faculty of Science and Agriculture, University of Fort Hare, Alice, South Africa; 2 Department of Agronomy, University of Fort Hare, Alice, South Africa; Public Health Agency of Canada, CANADA

## Abstract

Chikungunya virus (CHIKV) is an emerging arbovirus that causes major morbidity in tropical regions, but its epidemiology in Southern Africa is poorly defined. This study compiles research on CHIKV circulation, clinical characteristics, and surveillance strategies in the region, specifically in the Southern African Developing Community (SADC) block. A systematic search of peer-reviewed publications published between January 2012 and October 2025 that reported CHIKV detection, seroprevalence, or clinical symptoms in eight Southern African countries was conducted. Eligible studies included human studies, outbreak investigations, population-based surveys, diagnostic evaluations, and case reports. Whilst, zoonotic studies, animal studies, letters to editors, comments, and studies not clearly define in terms of country, number participants and method of investigation were considered ineligible. Twenty studies met inclusion criteria, representing data from Madagascar, Mozambique, the Democratic Republic of the Congo (DRC), Angola, Tanzania, Malawi, South Africa (SA), and Mauritius. CHIKV circulation was confirmed in both urban and rural settings, often co-occurring with dengue, Zika, and Rift Valley fever viruses. Seroprevalence estimates varied widely, from sporadic detection in Angola to sustained immunoglobulin G (IgG) positivity in Madagascar and Tanzania, indicating ongoing endemic transmission. Women of reproductive and working age were disproportionately affected. Clinical presentations were dominated by acute febrile illness with arthralgia, though severe neurological outcomes and long-term rheumatologic sequelae were reported. Diagnostic practices relied primarily on serology, with molecular confirmation limited to outbreak contexts. Vector surveillance detected CHIKV in mosquitoes even in the absence of human cases. CHIKV is a common virus in Southern Africa that is not well known. Effective response and early detection depend on strengthened integrated surveillance that combines entomological, molecular, and serological approaches. The findings highlight the necessity of investigating the long-term effects of Chikungunya infection and conducting coordinated regional monitoring.

## Author summary

The Chikungunya virus (CHIKV) is an arbovirus transmitted by mosquitoes, resulting in fever, intense arthralgia, and, in some cases, prolonged musculoskeletal disorders. Despite recurrent outbreaks in Africa, the epidemiology and public health burden of CHIKV in Southern Africa remain poorly understood. This systematic review synthesized studies published between January 2012 and October 2025 to examine the geographic distribution, reported occurrence, clinical manifestations, and surveillance challenges associated with CHIKV across the Southern African Development Community (SADC) region. The review identified evidence of CHIKV circulation in Madagascar, Mozambique, the Democratic Republic of Congo, Malawi, Tanzania, Angola, South Africa, and Mauritius. Outbreaks and serological evidence were reported across urban, rural, peri-urban, and coastal settings. Several studies also documented neurological complications and persistent musculoskeletal symptoms following infection. However, major differences in diagnostic methods, study designs, and surveillance capacity limited direct comparisons between countries. These findings highlight the urgent need for strengthened laboratory systems, harmonized surveillance, and integrated vector-control strategies to improve regional preparedness and public health response.

## 1. Introduction

Chikungunya virus (CHIKV) is a re-emerging alphavirus of the Togaviridae family, for which mosquitoes are the principal vector [1]. CHIKV have been classified into four distinct genotypes: West African, East/Central/South African (ECSA), Asian, and Indian Ocean lineages, with the latter being monophyletic descendants of the ECSA [2,3]. With the advent of nucleic acid sequencing and tools enabling the elucidation of molecular evolution, these CHIKV strains have been grouped and named according to their geographical origin [3]. CHIKV is transmitted by *Aedes* mosquitoes, causing a febrile illness with periodic outbreaks in large parts of the world [4]. It is associated with severe joint pains, rash, acute fever, severe arthralgia, headache, malaise, muscle, aches and retro-orbital pain [1,5]. Other authors have reported that it replicates in the skin, spread in the liver, lymphoid tissues and brain, through the blood [6]. Although reports of CHIKV-related fatalities are limited, they have occurred in several countries; for instance, in 2006, Reunion Island (775,000 inhabitants) recorded over 244,000 cases of CHIKV and 205 deaths directly or indirectly linked to the virus [7]. This corresponds to mortality rates ranging from 0.024 up to 0.7%, depending on both the viral genotype and neurological involvement [8]. In addition, CHIKV infection is associated with considerable morbidity, as arthralgia can be debilitating, and severe acute manifestations may progress to multi-organ failure and death [1]. While this virus is primarily transmitted by a wide range of mosquitos, maternal transmission resulting in neurologic and haemorrhagic complications in affected infants has also been reported [1].

The disease was first described during an outbreak in southern Tanzania in 1952 [4] and was later identified in 1959 in South Africa along with Usutu virus (USUV) [9]. Since then, CHIKV has caused large outbreaks affecting millions across Africa, South-East Asia and around the Indian Ocean [10]. Yellow fever and dengue mosquitos (*A. aegypti*), and Asian tiger mosquito (*A. albopictus*) are known as the main vectors worldwide, but lately, house (*Culex*) and malaria (*Anopheles*) mosquitoes have also been reported to transmit CHIKV in other countries, such as Kenya, Mozambique and South Africa [11]. In Africa, the virus is maintained in a sylvatic cycle involving forest-dwelling mosquitoes and non-human primates, with urban penetration and human-to-human transmission being fueled by two anthropophilic mosquitoes of the genus *Aedes*, specifically, *A. aegypti* and *A. albopictus* mosquitoes. Among these, *Aedes aegypti* is an urban mosquito responsible for most reported CHIKV transmissions worldwide [12], whereas *A. albopictus*, originally a zoophilic, forest-dwelling species from Asia, now exhibits a wider geographical distribution than *A. aegypti* [13].

CHIKV transmission has been widely reported in Africa in recent years, with endemic febrile disease being particularly prevalent in children [14]. CHIKV has been reported to be endemic in about 33 African countries, including Uganda, the DRC, Senegal, Republic of the Congo, Nigeria, Angola, Benin, Burundi, Cameroon, Central African Republic, Chad, Comoros, Cote d’Ivoire, Djibouti, Equatorial Guinea, Eritrea, Ethiopia, Gabon, Guinea, Kenya, Madagascar, Malawi, Mauritius, Mayotte, Mozambique, Réunion, Seychelles, Sierra Leone, South Africa, Somalia, Sudan, Tanzania, and Zimbabwe [15]. It has also been reported in Madagascar, Mozambique, Zambia, Kenya, Comoros, La Réunion, Mauritius, Seychelles, Mayotte, Cameroon, Tanzania, Gabon, DRC, Senegal, Angola, Malawi, and Mauritius [16,17]. Like in many other African countries, arthropod-borne diseases are a public health threat in the SADC. Although the CHIKV outbreak was first reported in southern Africa, alphaviruses have remained largely neglected in this region [17,18]. Out of all the countries within the Africa continent, Kenya has shown progress in terms of addressing alphaviruses outbreaks, especially, CHIKV. Emerging and re-emerging of CHIKV is currently a global public health concern due to its continued spread and escalating epidemic trends throughout the tropical and subtropical regions, particularly in sub-Saharan Africa where large epidemics have been observed [1,15]. Moreover, re-emergence of CHIKV is unpredictable, with intervals between consecutive epidemics ranging from 7 to 20 years. For example, from 1973 to 2005, no cases were reported in India and suddenly re-emerged of ECSA lineage as a major outbreak after 32 years in 2006, affecting 13 different states during 2005–06 period [19].

Currently, there are no licensed antivirals, therapeutic strategies, or vaccines available to remedy CHIKV [20]. Residents of Sub-Saharan Africa remain at high risk from several arboviral infections, particularly CHIKV, yet regional surveillance data are scarce, and the full burden of exposure and disease is largely unknown [17]. This systematic review aims to document the prevalence and status of CHIKV, a neglected disease in most SADC countries, through existing literature, providing insights into its epidemiology, disease burden, and consequences in the region. Specifically, the study addresses key questions, including: (i) What is the reported incidence and geographic distribution of CHIKV in Southern Africa? (ii) Which mosquito vectors are most frequently implicated in transmission? (iii) How do viral genotypes vary across outbreaks, and how are they linked to disease severity? (iv) Which populations are most affected, and what are the documented clinical outcomes and fatalities? Additionally, the review examines socio-economic factors influencing CHIKV transmission, diagnosis, and management, as well as the role of geographical variation in shaping virus transmission and maintenance.

## 2. Method

### 2.1. Search strategy and study selection

**Fig 1:**
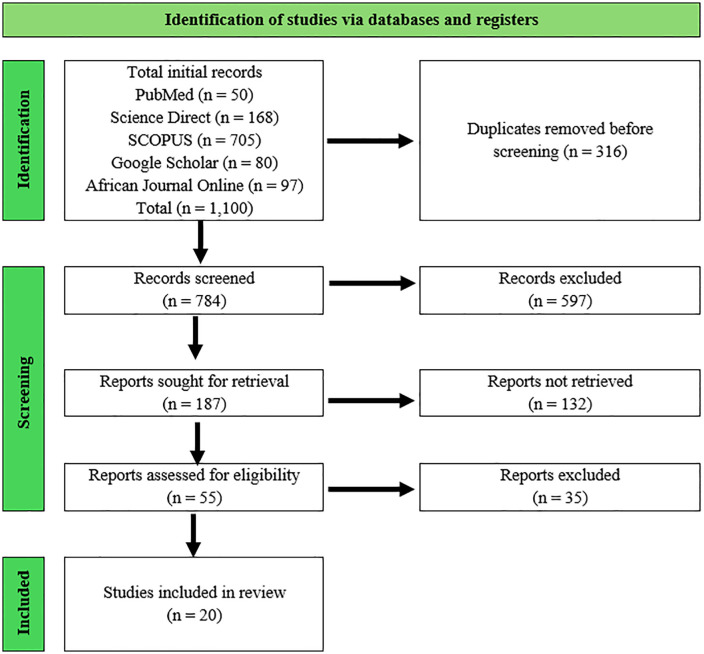
PRISMA flow diagram for eligible articles for inclusion in the systematic review.

A systematic search of literature published in English on CHIKV between January 2012 and October 2025 was meticulously carried out, adhering to the recommended methodology as per the guidelines presented in the Preferred Reporting Items for Systematic Reviews (PRISMA) as described by Brennan and Munn [21], elucidated in Fig 1. The review period (January 2012 to October 2025) was selected to capture contemporary evidence on CHKV epidemiology in the SADC region following the major global expansion between 2004 and 2011, while ensuring inclusion of the most recent studies reflecting evolving transmission dynamics, improved diagnostic capacity, and the increasing influence of climate and environmental change on arboviral spread. The study is registered with International prospective register of systematic reviews (PROSPERO): CRD420251250401 and the protocol can be found online https://www.crd.york.ac.uk/PROSPERO/view/CRD420251250401. All methodological procedures were predefined, documented, and followed to ensure transparency and reproducibility. A completed PRISMA 2020 checklist is provided in the supplementary materials (S1 File). This process involved the utilization of various search engines, including Scopus, PubMed, Science Direct, Google Scholar, and African Journal Online (AJO), all of which functioned as search databases for the collection and thorough analysis of data. A carefully curated set of keywords, detailed in Table 1, was employed to facilitate the precise retrieval and evaluation of relevant articles. The search focused on countries within the SADC region, as these countries share regional health policies, vector ecology similarities, and cross-border disease dynamics, which are particularly relevant for arboviral surveillance and control. The following set of keywords was used: {Health condition} “Chikungunya” OR “Chikungunya virus” OR “Chikungunya fever” OR “*Aedes aegypti*” OR “*Aedes albopictus*” OR “*Culex*” OR “*Anopheles*” AND “Prevalence” OR “Transmission” OR “Rate” OR “Occurrence” AND {Southern African Country} “Angola” OR “DRC” OR “Botswana” OR “Lesotho” OR “Malawi” OR “Mozambique” OR “Namibia” OR “Tanzania” OR “South Africa” OR “Swaziland” OR “Zambia” OR “Zimbabwe” AND “Human”. The keywords were carefully selected to aid in the precise retrieval and evaluation of relevant articles. The search returned 1,100 articles from Science Direct, Google Scholar, Scopus, PubMed, and AJO, respectively. The search was restricted to publications in the English language. This restriction was applied due to resource constraints and the need to ensure accurate interpretation but may have resulted in the exclusion of relevant studies published in other languages.

**Table 1:** Search strings used in this review.

Source	Search string	Search fields	Number of papers/articles
PubMed	((“Chikungunya” OR “Chikungunya virus” OR Chikungunya fever OR “Alphavirus” OR “*Aedes aegypti*” OR “*Aedes albopictus*” OR “*Culex*” OR “*Anopheles*” AND (“Prevalence” OR “Transmission” OR “Rate” OR “Occurrence”) AND (“Angola” OR “DRC” OR “Botswana” OR “Lesotho” OR “Malawi” OR “Mozambique” OR “Namibia” OR “Tanzania” OR “South Africa” OR “Swaziland” OR “Zambia” OR “Zimbabwe”)) AND “Human”. [MeSH terms]	Title, Abstract, MeSH terms	50
ScienceDirect	TITLE-ABS-KEY (**“**Chikungunya” OR “Chikungunya virus” OR Chikungunya fever OR “Alphavirus” OR “*Aedes aegypti*” OR “*Aedes albopictus*” OR “*Culex*” OR “*Anopheles*”) AND (“Prevalence” OR “Transmission” OR “Rate” OR “Occurrence” AND “Angola” OR “DRC” OR “Botswana” OR “Lesotho” OR “Malawi” OR “Mozambique” OR “Namibia” OR “Tanzania” OR “South Africa” OR “Swaziland” OR “Zambia” OR “Zimbabwe” AND “Human”)	Title, Abstract, Keywords	168
Google Scholar	**“**Chikungunya” OR “Chikungunya virus” OR Chikungunya fever OR “Alphavirus” OR “Aedes aegypti” OR “Aedes albopictus” OR “Culex” OR “Anopheles” AND “Prevalence” OR “Transmission” OR “Rate” OR “Occurrence” AND “Angola” OR “DRC” OR “Botswana” OR “Lesotho” OR “Malawi” OR “Mozambique” OR “Namibia” OR “Tanzania” OR “South Africa” OR “Swaziland” OR “Zambia” OR “Zimbabwe” AND “Human”	All fields (title, abstract, full text)	80
Scopus	TITLE-ABS-KEY (“Chikungunya” OR “Chikungunya virus” OR Chikungunya fever OR “Alphavirus” OR “*Aedes aegypti*” OR “*Aedes albopictus*” OR “*Culex*” OR “*Anopheles*” AND “Prevalence” OR “Transmission” OR “Rate” OR Occurrence AND (“Angola” OR “DRC” OR “Botswana” OR “Lesotho” OR “Malawi” OR “Mozambique” OR “Namibia” OR “Tanzania” OR “South Africa” OR “Swaziland” OR “Zambia” OR “Zimbabwe”) AND (“Human”).	Title, Abstract, Keywords	705
AJOL	“Chikungunya” OR “Chikungunya virus” OR Chikungunya fever OR “Alphavirus” OR “*Aedes aegypti*” OR “*Aedes albopictus*” OR “*Culex*” OR “*Anopheles*” AND “Prevalence” OR “Transmission” OR “Rate” OR “Occurrence” AND “Angola” OR “DRC” OR “Botswana” OR “Lesotho” OR “Malawi” OR “Mozambique” OR “Namibia” OR “Tanzania” OR “South Africa” OR “Swaziland” OR “Zambia” OR “Zimbabwe” AND “Human”	All fields (title, abstract, full text)	97

AJOL: African Journal Online; DRC: Democratic Republic of Congo.

### 2.2. Inclusion and exclusion criteria

The search was confined to peer-reviewed publications in the English language, with each article subject to rigorous evaluation against predetermined eligibility criteria (Table 2). Studies published between January 2012, and October 2025 were eligible for inclusion, irrespective of the exact period during which primary data collection occurred. Consequently, some included studies reported outbreak or surveillance data collected prior to 2012 but were retained because their findings were published within the predefined review timeframe. These criteria included the following: (a) original research articles originating from the SADC region, (b) epidemiological studies, clinical studies, vector studies, case reports and case series, surveillance and monitoring reports, intervention studies, genetic and molecular studies, policy and public health reports, and qualitative studies involving human subjects, (c) availability of the complete full-text document, and (d) a clear indication of the study’s geographical location. Notably, papers presenting primary data on the prevalence, economic burden, costs associated with interventions, or implications for health systems in the context of control and elimination programmes were selected for a more in-depth review. Additionally, papers featuring primary data on the costs related to any facet of treatment, prevention, or control were also included. The exclusion criteria encompassed, duplicates, unavailable full texts, or abstract-only papers, research done on non-southern African communities, review articles, letters, comments to the editor, and animal studies.

**Table 2:** Inclusion and exclusion criteria for studies considered in the systematic review on the incidence of CHIKV in Southern Africa.

Criteria	Inclusion	Exclusion	Rationale
Geographical scope	Studies conducted in SADC countries (Angola, Botswana, Comoros, DRC, Eswatini, Lesotho, Madagascar, Malawi, Mauritius, Mozambique, Namibia, Seychelles, South Africa, Tanzania, Zambia, Zimbabwe)	Studies outside the SADC region	Ensures relevance to the regional CHIKV epidemiology and vector distribution
Study design/ type	Epidemiological studies (cross-sectional, cohort, case-control), clinical studies, vector studies, case reports and case series, surveillance and monitoring reports, intervention studies, genetic and molecular studies, policy and public health reports, qualitative studies involving humans	Reviews, editorials, opinion pieces, meta-analyses, modelling-only studies without original data	Focuses on original research providing primary data relevant to CHIKV
Population/ subjects	Human participants of any age/sex; vector studies involving Aedes spp. mosquitoes from SADC countries	Animal-only studies (unless vectors are included); in vitro studies without human/vector relevance	Ensures data reflect CHIKV transmission, clinical outcomes, and vector ecology
Publication type/ availability	Original research articles, reports, and documents with full-text available in English (or translated)	Abstracts only, conference proceedings without full text, non-English publications without translation	Full-text access is required for reliable data extraction and quality assessment
Timeframe	Studies published from 2012 to 2025	Studies outside this timeframe	Captures both historical and contemporary evidence of CHIKV incidence and spread
Geographical clarity	Clear indication of study location within the SADC region	Studies lacking clear geographic information	Necessary for accurate mapping of incidence, prevalence, and vector distribution
Data relevance	Reporting CHIKV-related outcomes such as incidence, prevalence, clinical features, mortality, vector identification, viral genotypes, or public health interventions	Studies not reporting CHIKV-related outcomes	Focuses the review on data that directly inform disease burden and epidemiology

### 2.3. Study characteristics and data extraction

Upon identifying studies that met the inclusion criteria, a systematic record search was established, and a dedicated spreadsheet was generated to systematically organize and collate the pertinent information derived from these research articles. The spreadsheet meticulously catalogued the following details: author names, study titles, publication year, country of origin, gender, socio-economic classification (comprising low, middle, or high), residential status (urban or rural), educational background, population size under study, research findings, and study outcomes. However, many primary studies did not consistently report variables such as socio-economic status, education level, or place of residence. In such cases, data were recorded as “not reported” and no assumptions or imputations were made. When information was partially reported or ambiguous (e.g., broad age ranges, unclear case definitions, mixed populations), the most conservative interpretation was applied, and clarifications were based strictly on the published text. Studies were not excluded due to missing data. All extracted variables and missing fields are transparently reflected in the evidence tables (Tables 2,3), in accordance with PRISMA 2020 guidelines.

**Table 3:** Characteristics of all the eligible studies conducted in Southern Africa countries on Chikungunya virus.

S/N Country	Aim of the study	Age group	Gender	Socio-economicclass (Low,middle, or high)	Place of residence (Urban or rural)	Educational status	Population size	Summary of findings	Reference
Madagascar	The objective of the current study was to estimate the seroprevalence of these three arboviruses in different areas of Madagascar through serum samples obtained in populations surrounding the SHC network in all regions of the country and analyzed for the presence of IgG antibodies.	≥18 years	(50.7%); females	Low-income	Rural, suburban and urban	Less to moderate	1680 individuals	All the three viruses were found to be actively circulating in Madagascar before or during the study period	[23]
Madagascar	To estimate the prevalence of IgG antibodies against six arboviruses in PLWHIV in Madagascar	0-55 years	50% female; 50% males	Low-income	Rural-urban	Less to moderate	1036 samples tested	The study found high seroprevalence of IgG antibodies to the two alphaviruses of (CHIKV and O’nyong’nyong virus (ONNV), WNV, DENV 1, and, to a lower level, USUV and DENV-3.	[9]
Madagascar	This study aims to evaluate the use of capillary blood samples blotted on filter papers for molecular diagnosis of CHIKV infection.	≥18 years	Sex ratio: (M/F) were 1.2 and 0.5, respectively	Low-income	Rural-urban	Less to moderate	181 patients tested	The study showed that for diagnosis, outbreak monitoring and virological surveillance of CHIKV infection and circulation, capillary blood samples taken from the finger and spotted onto filter paper is a cost-effective alternative with a good sensitivity and specificity (93.1% and 94.4%, respectively)	[16]
Madagascar	To assessed the serologic markers and reported clinical features of women who came for routine pregnancy follow-up visits at 6 geographic locations.	12–50 years	100% females	Low-income	Rural coastal to semi-urban	Less to moderate	1,244 women	The study found the outbreak in this case to be exclusively caused by CHIKV infections without concomitant DENV infection.	[26]
Angola	To conduct epidemiological and biological investigations to identify the etiology of these cases to contribute to better management of AFP in Kinshasa.	12–41 years	Females 34 (59.6%); Males 24 (40.4%)	Low-income to high income	Rural	Less educated	57 patients	No virus material was found, however the serological test (ELISA) revealed antibodies to CHIKV, 47.4% (27/57) for IgM and 22.8% (13/57) for IgG. Among suspected cases, we found anti-Chikungunya IgM in 33.3% (7/21) and anti-Chikungunya IgG in 14.3% (3/21).	[27]
Angola	To analyze clinical, demographic and epidemiological data from 351 suspected dengue cases sampled across 13 provinces in Angola, including cases of severe dengue in Angola.	<18 years	Male (60%	Low to middle income	Rural to urban	Less educated	351 serum (blood) samples	This study described characteristic of predominately urban transmission of DENV2 in Angola and also co-circulation of DENV2 with DENV1 and CHIKV.	[31]
DRC	The aim was to investigate the outbreak in order to confirm CHIKV as the causal pathogen, to assess the epidemiological, clinical, laboratory, and entomological characteristic of the outbreak and to gain knowledge on the perception of the population regarding the outbreak.	≥10 years	Female (61.8%)	Low to high income	Urban	Moderate to well educated	2686 suspected cases	The findings indicated a large outbreak and no/or limited to prior exposure to chikungunya virus in the investigated areas.	[22]
DRC	To molecularly characterize the CHIKV outbreak that hit in Kinshasa, Kasangulu, and Matadi, DRC in early 2019	Randomized	Randomized	Low to high income	Urban area	Moderate to well educated	175 patients	The study was able to detect and sequence the virus in both human and mosquito samples and confirmed that the circulating virus belonged to the ECSA lineage.	[13]
DRC	To quantify the importance of four major arboviruses as a cause of acute undifferentiated fever and to describe the case presentation and the presence of arbovirus/malaria co-infections	≥2 years	183 (53.5%) female	Middle to high income	Peri-urban	Moderate to well educated	342 patients	Acute DENV, caused by DENV1 and DENV2, and CHIKV infection was demonstrated in 8.1% and 0.9% of the patients attending a first line health center with acute undifferentiated fever, respectively.	[33]
Mozambique	Assessing the occurrence and trends of arboviruses (dengue, Zika, chikungunya) and rodent-borne diseases’ (leptospirosis) transmission in the aftermath of cyclones Idai and Kenneth,	≥15 years	58.4%; women	Low-income	Rural, semi-urban	Less to moderately educated	305 patients	104 (34.1%) patients tested showed specific IgM antibodies against DENV, ZIKV, CHIKV or *Leptospira*.	[30]
Mozambique	Seroepidemiology of CHIKV Among Febrile Patients in Eight Health Facilities in Central and Northern Mozambique, 2015–2016	<23 years	45.7% were female	Low-high income	Rural-urban	Less to highly educated	392 patients	The frequency of participants with seropositivity for IgM and IgG anti-CHIKV antibodies was 1.5% (6/392) and 28.6% (112/392), respectively. Patients with seropositivity for IgM anti-CHIKV were significantly younger.	[34]
Mozambique	The first case of CHIKV disease with a severe clinical course from an adult patient in Pemba, Mozambique.	≥40 years	1 Male	Low-income	Rural	Less educate	1 patient	This report suggests that CHIKV may be associated or cause unsuspected severe disease in febrile patients in Mozambique.	[24]
Mozambique	To provide recent information on the frequency of CHIKV in acute febrile patients in southern Mozambique.	> 5 years	57.5% (224/391) female	Middle to high income	Suburban	Moderated to well educated	391 patients	The study showed that the clinical presentation of patients with acute CHIKV infection was similar of that from patients with previous exposure or with no CHIKV infection.	[29]
Mozambique	The aim of this study was to investigate the occurrence of CHIKV during an outbreak of DENV in Pemba city in northern Mozambique in 2014.	20–34 years	52.7% (77/146) male and females	Middle-high income	Urban	Moderate to well educated	146 participants	The finding suggested that Mozambicans had been silently exposed to the DENV but also CHIKV and the viruses needs to considered in febrile patients seeking medical attention	[28]
Tanzania	This study was conducted to investigate the prevalence of DENV and CHIKV in Kilombero Valley, Tanzania.	≥7 years old	females (65%)	Low-income	Rural	Less educated	294 patients	This study revealed the co-circulation of all 4 DENV serotypes and CHIKV across all age groups during both the dry and rainy seasons in Kilombero.	[32]
Tanzania	This study aimed to determine dengue and chikungunya’s seroprevalence in blood donors using the stored blood samples from Temeke referral hospital in Dar es Salaam and Zanzibar National Blood Bank in Zanzibar.	< 30 years	95% male	Low to average income	Rural to urban	Less to high educated	281 blood samples	CHIKV IgG seropositivity significantly higher in Dar es Salaam than Zanzibar; IgM detected indicating recent infection; Dual IgG suggests co-exposure. Lower CHIKV IgG seropositivity and no IgM detected, suggesting lower or earlier transmission.	[35]
Tanzania	To determine seroprevalence of CHIKV among febrile patients seeking medical care at health facilities in Karagwe, Sengerema, Kilombero and Kyela districts.	> 1 year	266 (36.5%) males; 462 (63.5%) females	Low-income	Rural	Less to moderately educated	1,310 patience’s; 728 (55.6%) met the study criteria	The seroprevalence of chikungunya among febrile participants was found to be the highest reported in Tanzania.	[1]
South Africa	The aim of the study was to obtain current data regarding alphaviruses circulating in the north-eastern provinces of South Africa, where most neurological cases were identified in humans and animals and identify the potential associated vectors.	–	–	Low to high income	urban, peri-urban, rural, and conservation areas	Less to highly educated	64,603 adult mosquitoes belonging to 11 genera	During the study period, no CHIKV-positive pools were found, despite previous outbreaks in Northern KwaZulu-Natal and Eastern Limpopo Provinces. The trap type used here did not target *Aedes furcifer*, the mosquito species that was responsible for CHIKV transmission in the past.	[18]
Malawi	To determine the seroprevalence of CHIKV and to molecularly confirm the presence of CHIKV RNA among febrile outpatients seeking health care at Mzuzu Central Hospital in the Northern Region of Malawi.	2–83 years	79 (66.39%) females; 40 (33.61%) males.	Middle to high income	Urban	Moderate to well educated	119 participants	Anti‑CHIKV IgM seroprevalence 61.3% among 119 suspected samples; confirmed CHIKV RNA detection in selected samples.	[25]
Mauritius	assessing the prevalence of and risk factors for chronic musculoskeletal symptoms and for a rheumatoid arthritis-like condition at 27.5 months after initial infection.	>18 years	30 males; 143 females	High income	Semi-urban	Highly educate	195 participants	The findings from this study suggested that CHIKV causes significant long-term joint pains and disability	[10]

The evaluation of the data’s relevance predominantly relied on an initial assessment of the tittle, followed by the abstracts, and a thorough examination of the full-text articles. This process was initiated with an initial screening of titles and abstracts to gauge their conformity with the eligibility and relevance criteria established for this systematic review, following the pre-defined inclusion and exclusion criteria. In the course of this review, any books scrutinized were limited to those containing independently published papers that had subsequently been consolidated as book chapters. Additionally, to maintain the integrity of the review, duplicate studies were meticulously identified and subsequently expunged from the pool of selected research articles. The chosen papers were retrieved, read for data extraction, and subjected to a comprehensive review of the full-text content. The extracted data were diligently recorded in a structured tabular format for systematic analysis.

### 2.4. Publication bias and limitations

In this systematic review, we aimed to synthesize the available literature on the reported prevalence CHIKV in SADC countries based on the predefined inclusion criteria. Study quality and risk of bias were independently assessed by two reviewers using the Joanna Briggs Institute (JBI) Critical Appraisal Checklist for Prevalence Studies. Discrepancies in scoring were resolved through discussion and consensus. Each study was evaluated according to sampling methodology, diagnostic confirmation, population representativeness, and completeness of reporting, and subsequently categorized as having low, moderate, or high risk of bias based on the number of domains adequately fulfilled (low = 7–9 criteria met; moderate = 4–6; high = ≤3). Study screening followed a standardized two-stage approach. Two reviewers independently screened titles and abstracts, followed by independent full-text assessment of potentially eligible articles. Any disagreements were resolved through discussion, and unresolved discrepancies were adjudicated by a third reviewer. Inter-reviewer agreement during screening was quantified using Cohen’s kappa statistic to ensure methodological rigor. A PRISMA 2020 flow diagram summarizing study identification, screening, eligibility assessment, and final inclusion is presented in Fig 1. Despite comprehensive searching, the availability of CHIKV prevalence data from SADC countries was limited, which posed challenges in synthesizing evidence and highlights the need for strengthened surveillance and reporting systems in the region. Furthermore, this review was limited to English-language publications, which may have excluded relevant studies from Lusophone and Francophone SADC countries such as Mozambique, Angola, and the Democratic Republic of Congo. This introduces potential language bias and may have influenced the comprehensiveness of the evidence synthesized.

**Fig 2:**
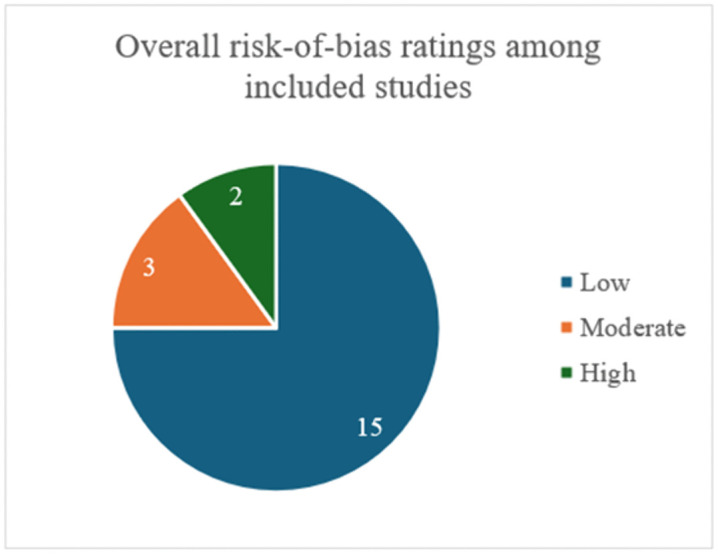
Summary of overall risk-of-bias ratings among included studies.

Fig 2 displays the percentage distribution of the 20 studies across three overall risk-of-bias categories, based on the Joanna Briggs Institute Critical Appraisal Checklist for Prevalence Studies. Studies meeting 7–9 criteria were classified as low risk, 4–6 criteria as moderate risk, and ≤3 criteria as high risk. Percentages were calculated relative to the total number of included studies (N = 20).

## 3. Results

### 3.1. Characteristics of included studies

Twenty studies published between January 2012 and October 2025 were identified across eight countries in Southern Africa, namely Madagascar, Mozambique, the DRC, Angola, Tanzania, Malawi, South Africa, and Mauritius (Table 3). Although the review inclusion criteria were based on publication date, several studies analyzed outbreaks or surveillance datasets collected prior to 2012, particularly retrospective investigations and longitudinal follow-up studies. The studies employed a wide range of designs, including large-scale outbreak investigations [1,22], population-based surveys [1,23], diagnostic evaluations [16], and case reports [24]. Sample sizes varied substantially across studies. Some studies reported individual case descriptions [24], while others included large cohorts, such as 2,686 suspected cases reported in the DRC [22]. Several studies recruited participants from outpatient clinics or community-based settings, with sample sizes ranging from fewer than 100 to over 1,000 individuals.

The populations studied differed across settings. Community-based seroprevalence studies were conducted in Madagascar [23], while health facility–based investigations in Tanzania [1] and Malawi [25] focused specifically on febrile outpatients. Specific subpopulations included pregnant women in Madagascar [26], people living with human immunodeficiency virus (PLWHIV) [9], and patients with neurological complications in Angola [27]. Participant demographics varied across studies. Some studies captured broad age ranges, such as 2–83 years in Malawi [25], while some studies included only adults [23] or specific groups such as women of reproductive age [26]. Gender distribution was approximately balanced in several studies; however, higher proportions of female participants were observed in Tanzania (63.5% female; [1]) and the DRC (61.8% female; [22]). Socio-economic and educational characteristics were inconsistently reported. Most studies were conducted in low- to middle-income settings, particularly in rural and peri-urban areas of Madagascar, Tanzania, and Mozambique. One study from Mauritius included participants from a higher-income and more highly educated population [10].

**Fig 3:**
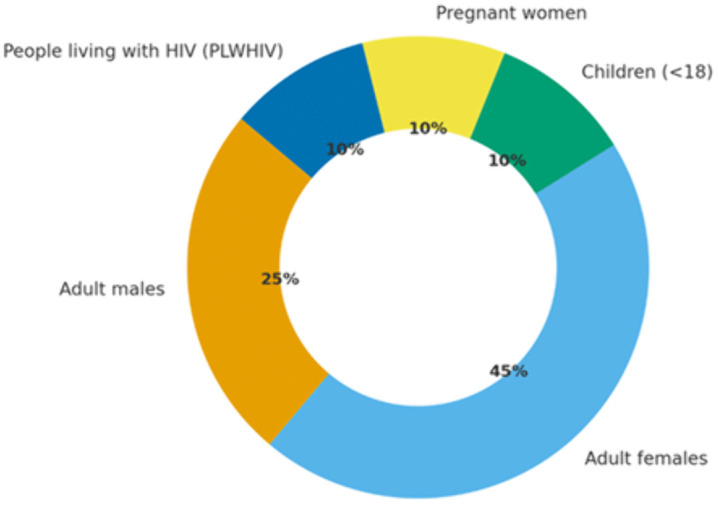
Representation of study populations across included chikungunya virus (CHIKV) studies in the Southern African Development Community.

Fig 3 summarizes the representation of study populations across the included studies (n = 20), based on demographic information reported in 18 studies. The proportions shown are derived from study-level reporting rather than pooled participant-level data. Population categories are not mutually exclusive (e.g., pregnant women are a subset of adult females, and people living with human immunodeficiency virus [PLWHIV] may span multiple age and sex groups) and therefore overlap may occur. Adult female populations were most frequently represented, followed by adult males, while children, pregnant women, and PLWHIV were also included in several studies. These proportions are indicative only and reflect the distribution of research focus and study sampling rather than the underlying population-level burden of CHIKV infection (synthesized from [1,9,10,16,22,23,26,27]).

### 3.2. Geographic and epidemiological distribution

**Fig 4:**
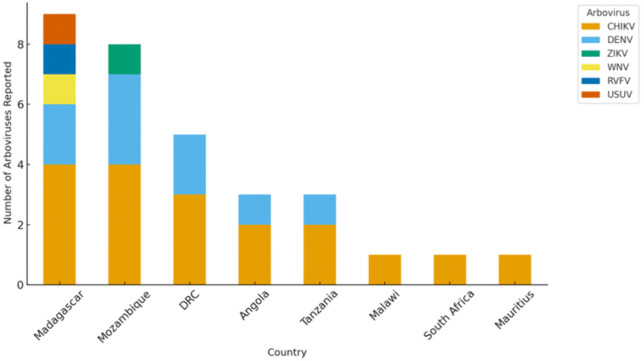
Arboviruses reported in included studies across countries of the Southern African Development Community (SADC) (publication period: January 2012–October 2025).

Across the included studies, CHIKV was reported in a range of geographic settings, including urban, peri-urban, rural, and coastal areas. Evidence of CHIKV circulation was reported in Madagascar from both population-based surveys and outbreak investigations [9,16,23,26]. In Mozambique, CHIKV was detected during dengue fever outbreaks [28,29] to post-cyclone transmission in flood-affected communities [30]. In the DRC, multiple large-scale outbreaks were reported, particularly in Matadi, Kinshasa and Kasangulu provinces, with a total of 2,686 suspected cases reported in one study [22]. Molecular analysis confirmed the circulation of the East/Central/South African (ECSA) lineage [13]. In Angola, serological evidence of CHIKV infection was reported among patients presenting with neurological symptoms [27], and co-circulation with dengue virus (DENV) serotypes was documented [31]. In Tanzania, CHIKV was reported alongside all four DENV serotypes in Kilombero Valley [32]. In Malawi, both serological and molecular evidence of CHIKV infection were identified among febrile patients [25]. In South Africa, CHIKV was detected in mosquito populations, although no human cases were confirmed in the included study [18]. In Mauritius, post-outbreak investigations reported persistent musculoskeletal symptoms among individuals with clinically suspected prior chikungunya infection, although long-term laboratory confirmation was not performed [10]. However, these findings should be interpreted cautiously because symptoms may overlap with other endemic infectious diseases and chronic conditions. Several included studies additionally reported the presence of other arboviruses, including DENV, Zika virus (ZIKV), West Nile virus (WNV), and Rift Valley fever virus (RVFV). However, because this review specifically focused on CHIKV-related investigations, the included evidence was not designed to comprehensively evaluate arboviral co-circulation across the SADC region. Consequently, these findings should be interpreted cautiously [9,10,18,23,25,30]. Fig 4 provides a descriptive summary of arboviruses reported within the included studies only, and reflects study-level observations rather than a comprehensive regional arbovirus surveillance analysis

The y-axis represents countries included in the review, while the x-axis indicates arboviruses reported in at least one included study from each country. Colored segments denote reported occurrence (presence/absence) only and do not represent incidence, prevalence, frequency of detection, or confirmed co-infection. The figure summarizes findings from CHIKV-focused investigations and should not be interpreted as a comprehensive regional assessment of arboviral transmission dynamics or co-circulation.

### 3.3. Seroprevalence and incidence findings

**Fig 5:**
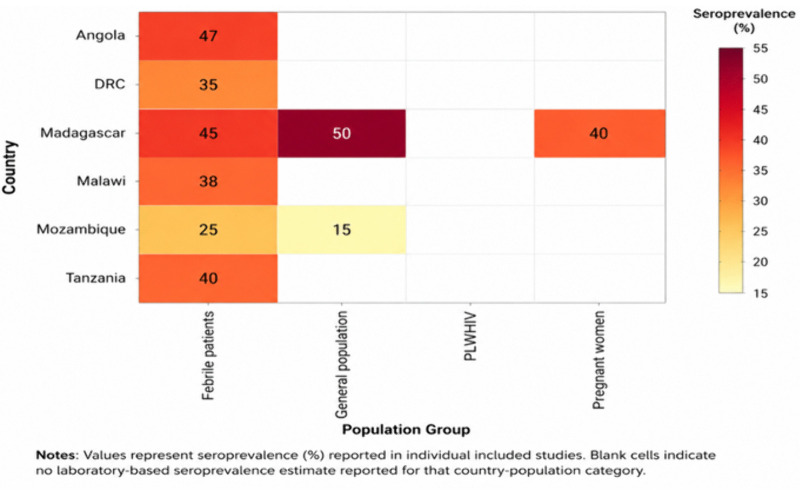
Study-level laboratory-based seroprevalence estimates of chikungunya virus (CHIKV) across selected countries and population groups in Southern Africa (publication period: January 2012–October 2025).

Seroprevalence estimates and incidence reports highlighted substantial heterogeneity in exposure across countries and study populations. In Madagascar, population-based surveys revealed widespread IgG seropositivity, indicating sustained exposure in both general communities and among vulnerable groups such as PLWHIV [9,23]. Among pregnant women, outbreak investigations confirmed CHIKV as the primary etiological agent responsible for a febrile illness cluster [26]. In the DRC, outbreak investigations documented large numbers of suspected CHIKV cases, including more than 2,600 suspected infections reported during a major outbreak [22]. Molecular investigations further confirmed circulation of the East/Central/South African (ECSA) lineage in both human and mosquito samples [13]. A smaller facility-based study in Kinshasa detected acute CHIKV infection in nearly 0.9% of patients presenting with undifferentiated fever [33]. Fig 5 presents a descriptive summary of laboratory-based seroprevalence estimates reported across selected countries and study populations. Estimates are grouped into broad categories (low < 10%, moderate 10–40%, and high >40%) to illustrate reported patterns of exposure. These findings should not be interpreted as directly comparable prevalence estimates because included studies differed substantially in sampling design, diagnostic assays, study setting, case definitions, and target populations.

In Tanzania, seroprevalence was reported among febrile patients attending healthcare facilities [1], and co-circulation with DENV was documented [32]. In Mozambique, serological studies reported both IgM and IgG antibodies among febrile patients [28–30,34]. In Malawi, both serological and molecular evidence confirmed CHIKV infection among febrile outpatients across a broad age range (2–83 years) [25]. In Angola, IgM and IgG antibodies were detected in 47.4% and 22.8% of tested individuals, respectively, although these findings were derived from a relatively small clinical cohort and should therefore be interpreted cautiously [27]. Because of substantial methodological heterogeneity across studies, Fig 5 is intended solely as an illustrative summary of study-level laboratory findings rather than a standardized comparison of CHIKV seroprevalence across Southern Africa. Mauritius-derived values and non-laboratory-confirmed estimates were excluded from Fig 5 to improve methodological consistency and reduce the risk of overinterpretation.

### 3.4. Clinical manifestations and outcomes

Clinical presentations of CHIKV infection were reported across multiple studies. Commonly reported symptoms included acute febrile illness and arthralgia [1,29,33]. In several settings, clinical presentations overlapped with those of other febrile illnesses such as malaria and dengue [1,29,33]. In Angola, patients aged 12–41 years presented with lower limb paralysis, with women more commonly affected than men [27]. In Mozambique, a severe case requiring hospitalization was described in an adult patient [24]. Studies involving pregnant women in Madagascar reported CHIKV infection among individuals attending antenatal clinics [26]. Post-outbreak investigations in Mauritius described persistent musculoskeletal symptoms among individuals with clinically suspected CHIKV infection identified during the 2006 epidemic; however, these findings should be interpreted cautiously because diagnoses were not laboratory confirmed and relied on retrospective self-reported symptom histories [10].

### 3.5. Diagnostic and surveillance approaches

The included studies employed a wide array of diagnostic and surveillance approaches, reflecting differences in resources, study aims, and epidemiological context. Serological assays, including IgM and IgG Enzyme-Linked Immunosorbent Assay (ELISA), were commonly used to detect acute and past infections [1,23,27]. While Molecular diagnostic techniques, including reverse transcription polymerase chain reaction (RT-PCR) and genomic sequencing, were used in outbreak investigations and research settings [13,25]. These methods enabled detection of viral RNA and identification of circulating lineages. Alternative sampling methods were also reported. In Madagascar, dried blood spot sampling demonstrated sensitivity of 93.1% and specificity of 94.4% for CHIKV detection [16]. In addition to human diagnostics, entomological surveillance was conducted in South Africa, where CHIKV was detected in mosquito populations [18]. The reported approaches varied across studies in terms of diagnostic methods, sample types, and surveillance strategies.

## 4. Discussion

This rigorous synthesis of information from Southern Africa demonstrates that CHIKV is geographically broad and epidemiologically diverse, indicating its return as a regional public health issue. Transmission was documented between 2012 and 2025 from coastal islands like Mauritius and Madagascar to interior and highland areas in Malawi, Tanzania, and the DRC. The intermittent nature of outbreaks, combined with changing research capability, is mirrored in the variety of study designs, which include outbreak investigations, population-based surveys, and diagnostic evaluations. These findings improve our understanding of CHIKV epidemiology, clinical presentation, and diagnostic problems in Southern Africa [36,37].

Crucially, the observed heterogeneity draws attention to critical gaps in the infrastructure for surveillance and diagnosis. Serological tests with variable sensitivity and specificity were used in many investigations, which limited comparability and might have underestimated true prevalence. Furthermore, there are a lot of unknowns about endemic transmission patterns, long-term immunity, and vector ecology in inland and highland areas due to the prevalence of outbreak-focused studies. These restrictions make it more difficult to identify outbreaks in a timely manner, estimate risks, and create focused intervention plans from a public health standpoint. Despite these challenges, the synthesis emphasizes the urgent need for integrated surveillance systems that combine molecular diagnostics, seroepidemiology, and entomological monitoring. Strengthening regional laboratory capacity and harmonizing study methodologies would facilitate more accurate incidence estimates, improve outbreak preparedness, and guide resource allocation. Furthermore, understanding the sociocultural and ecological factors that drive CHIKV transmission in diverse Southern African settings remains a critical research priority. Collectively, addressing these knowledge gaps will be essential for mitigating CHIKV’s public health impact and informing regional arboviral control strategies.

### 4.1. Geographic and epidemiological insights

The study findings suggest that CHIKV is geographically widespread and epidemiologically heterogeneous across Southern Africa, with Madagascar, Mozambique, and the DRC identified as persistent hotspots due to ecological conditions favorable for the primary vectors, *Aedes aegypti* and *Aedes albopictus* [13]. Genomic analyses indicate that the ECSA lineage predominates, with continuity observed in the DRC and Madagascar [13]. Recent detections in Malawi [25] and Angola [27] expand the recognized distribution beyond traditional coastal and tropical zones. Although direct incidence data remain sparse, outbreaks such as the DRC event of 1999–2000, estimated at over 50,000 cases, and regional seroprevalence studies reporting IgM and IgG prevalence of approximately 9.7% and 16.4%, respectively, indicate substantial underlying transmission, much of which likely remains undetected. The overlapping habitats of vectors, shared climatic drivers, and human mobility facilitate multi-pathogen transmission, highlighting the complex interplay of ecology, climate, and virus spread.

The limits of infrequent outbreak investigations and inadequate longitudinal surveillance have public health implications, as they hinder accurate endemic mapping and transmission dynamics prediction. Even previously low-risk areas may continue to see low-level, cryptic transmissions. Environmental changes, urbanization, and population movement may allow viruses to spread into higher-altitude or non-traditional zones, underlining the significance of proactive surveillance. Integrating genomic surveillance with epidemiological approaches can elucidate viral lineage distribution, improve epidemic attribution, and aid in the development of region-specific mitigation strategies. Comprehensive, standardized monitoring systems that monitor incidence, spatial distribution, and vector dynamics are critical for improving preparedness and targeting interventions. Integrating genomic surveillance with epidemiological methodologies can elucidate viral lineage distribution, improve outbreak attribution, and support development of region-specific mitigation strategies. Collectively, these findings underscore the urgent need for comprehensive, harmonized surveillance systems that track incidence, spatial distribution, vector ecology, and socio-demographic risk factors, thereby strengthening CHIKV preparedness and targeted public health interventions throughout Southern Africa.

### 4.2. Seroprevalence and demographic patterns

Due to methodological variations and actual epidemiological heterogeneity, seroprevalence estimates in Southern Africa exhibit significant variability. While high IgG seropositivity in Tanzania [1] and Madagascar [9,23] shows established endemicity, high IgM detection rates in Angola and the DRC imply recent introduction or re-emergence. These trends point to significant undetected transmission, especially in communities with little resources or those living in remote areas. Adults, particularly women of reproductive and working age, are disproportionately affected, most likely due to caregiving, occupational, and mobility-related exposures. Seropositivity spans all age groups, including children and the elderly, but chronic outcomes such as persistent arthralgia are more common in middle-aged women [10]. Socioeconomic factors, such as restricted access to healthcare, inadequate vector control methods, and rural living conditions, all influence exposure risk and can cause delays in diagnosis and treatment. Geographic variation, such as urbanization, altitude, and local ecological factors, influences virus survival, outbreak potential, and population risk. Integrating seroepidemiological, demographic, socioeconomic, and ecological data is thus essential for targeted surveillance and public health interventions. Targeted strategies should address the specific vulnerabilities of women of reproductive and working age, rural and low-income communities, and other groups at elevated risk, while strengthening surveillance, outbreak response, and vector-control programs to mitigate the public health burden of CHIKV across Southern Africa.

### 4.3. Clinical manifestations and long-term outcomes

CHIKV infection in Southern Africa is commonly characterized by acute febrile illness accompanied by severe polyarthralgia, consistent with global clinical descriptions of the disease [38]. Several included studies also reported severe or atypical manifestations, including persistent musculoskeletal symptoms following the Mauritius outbreak [10] and neurological manifestations among patients in Angola [27]. However, interpretation of these findings is limited by the cross-sectional design, reliance on self-reported clinical history, absence of laboratory confirmation, and potential selection and recall bias. In addition, overlapping symptoms with other endemic febrile illnesses such as dengue and malaria may have contributed to symptom misclassification. Therefore, the evidence for long-term CHIKV-associated morbidity in this setting remains suggestive rather than definitive. The prevalence of women among chronic cases suggests that immunological, hormonal, or occupational factors warrant additional investigation [20]. Clinical overlap with other febrile illnesses, particularly malaria and dengue, has been reported in Tanzania, Mozambique, and the DRC, often resulting in misdiagnosis [29,32]. While most cases are self-limiting, chronic morbidity can impose a significant health burden, particularly among vulnerable populations. These findings highlight the need for integrated clinical, epidemiological, and laboratory surveillance to guide accurate diagnosis, case management, and targeted public health responses.

### 4.4. Diagnostic and surveillance challenges

The examined studies repeatedly identified severe gaps in diagnostic capacity, suggesting significant challenges in the reliable identification of CHIKV in Southern Africa. Serological assays remain the primary diagnostic tool, but their specificity is limited by frequent cross-reactivity with other flaviviruses, such as dengue and Zika viruses [39,40]. While molecular techniques like RT-PCR and genomic sequencing provide greater sensitivity and specificity, their use is limited to research settings and outbreak investigations due to cost, infrastructure, and technical expertise requirements [13,25]. Novel strategies are being developed to overcome these limitations in regions with limited resources. For example, Madagascar has demonstrated that dried-blood spot sampling can be a practical substitute for CHIKV detection, allowing for more extensive surveillance without the need for complex laboratory equipment [16]. Simultaneously, entomological surveillance programs in South Africa serve as an excellent example of the benefits of implementing a One Health Approach, which integrate data on human diseases, vector populations, and environmental monitoring to better predict and address epidemics [18].

It is vital to develop standardized diagnostic procedures for a coordinated regional surveillance network. Such integration would not only improve early outbreak detection, but it would also provide critical insights into virus genotype variation, disease severity, and population-wide impact. Furthermore, extensive surveillance can enhance our understanding of clinical outcomes and mortality, inform targeted interventions, and strengthen public health preparedness. Finally, closing these diagnostic and surveillance gaps is critical to lowering the CHIKV burden and strengthening response strategies in Southern Africa.

### 4.5. Public health implications and future directions

According to the available data, Chikungunya is a disease that is underdiagnosed but is actively spreading throughout Southern Africa, frequently in combination with other arboviruses. To identify transmission dynamics and long-term health consequences, laboratory networks must be strengthened, vector control infrastructure must be improved, and expanding longitudinal cohort studies are necessary to define transmission dynamics and long-term health burdens. Climate unpredictability, increased urbanization, and population movement are expected to exacerbate arbovirus transmission in the next years, necessitating careful surveillance. Regional collaboration through platforms such as the Africa Centre for Disease Control and Prevention (Africa CDC) and the SADC health programs can facilitate timely data exchange, harmonized response strategies, and rapid outbreak containment. Future research priorities should include genomic surveillance of circulating CHIKV strains, vector competence studies, and evaluation of chronic sequelae to better inform clinical management and public health policy. By addressing these gaps, public health systems can anticipate outbreaks more effectively, mitigate disease impact, and guide evidence-based interventions across Southern Africa.

### 4.6. A “One Health” perspective for chikungunya surveillance and control in Southern Africa

**Fig 6:**
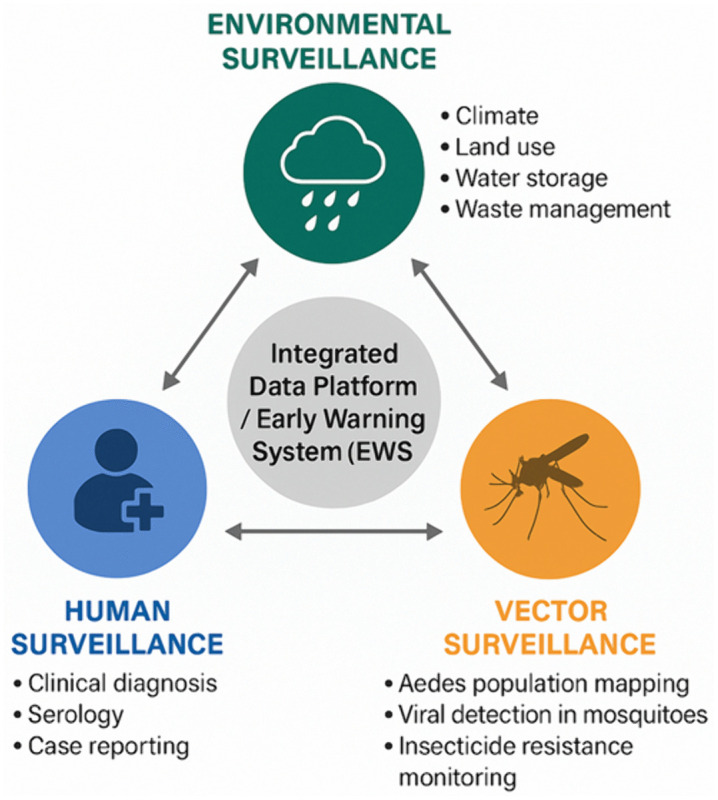
Conceptual “One Health” framework for chikungunya surveillance and control in Southern Africa.

The epidemiological patterns identified in this review characterized by recurrent outbreaks, undocumented inter-epidemic transmission, and heterogeneous diagnostic capacity highlighting the need for more integrated surveillance approaches [22,23]. A “One Health” perspective is particularly relevant because CHIKV transmission in Southern Africa arises from the interaction of human cases, *Aedes* vector ecology, and environmental drivers. Current systems remain predominantly reactive and fragmented, which limits early detection of circulation and delays coordinated response efforts (Fig 6).

Within the human health sector, the reviewed studies show substantial variability in diagnostic practices, from clinical-only case identification to PCR and serological confirmation. Integrating this data with systematic entomological surveillance would improve situational awareness, particularly where outbreaks were preceded by increases in vector density or detection of viral RNA in *Aedes* populations [13,18]. The environmental determinants documented across the included studies including rainfall anomalies, water-storage practices, and peri-urban breeding habitats further underscore the value of incorporating climate and land-use information into routine risk assessment [30,32].

**Fig 7:**
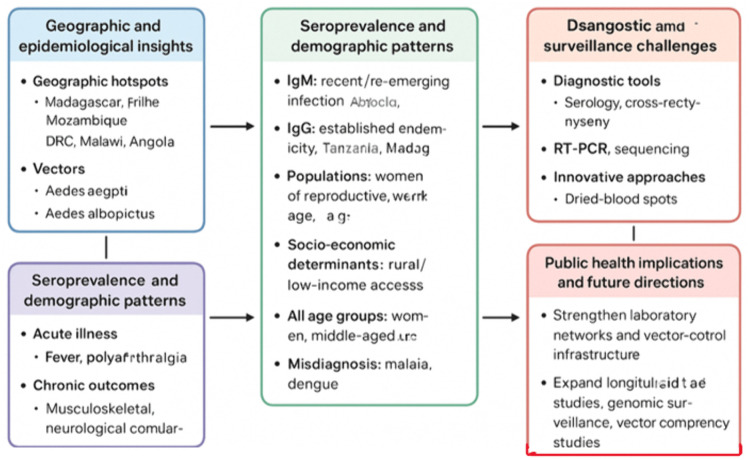
Integrated overview of CHIKV epidemiology, clinical Outcomes, and public health priorities in Southern Africa.

Therefore, to facilitate prompt detection of CHIKV activity, a streamlined One Health approach would prioritize data exchange amongst epidemiological, entomological, and environmental monitoring systems. By enhancing early warning capabilities and promoting evidence-based vector-control decision-making, such integration is consistent with more general regional health-security objectives. Although connections to sustainable development goals such as climate-informed planning (SDG 13) and health preparedness (SDG 3) are pertinent, their inclusion should be concentrated on how they directly improve CHIKV surveillance rather than expanding into broader conceptual commentary [9,27]. Fig 7 shows the integrated overview of CHIKV epidemiology, clinical Outcomes, and public health priorities in Southern Africa. Operationalizing these systems demands interoperable databases, decentralized diagnostics, and transparent information-sharing mechanisms across the SADC. Embedding One Health principles into CHIKV control strategies also advances sustainable development goal (SDG) 3 (Good Health and Well-Being) by shifting surveillance from reactive outbreak management to preventive, climate-responsive public-health preparedness. In doing so, Southern Africa can build resilient health systems capable of addressing not only CHIKV but also the broader spectrum of emerging vector-borne and climate-sensitive diseases. Overall, by enhancing coordination, facilitating proactive outbreak detection, and bolstering long-term resilience against CHIKV and associated arboviral threats in Southern Africa, using a One Health framework offers a practical mechanism to operationalize the review’s findings.

## 5. Limitations and prospects

Some limitations must be considered when interpreting the results of this systematic review. Direct comparison of seroprevalence estimates was made more difficult by the included studies’ heterogeneous designs, diagnostic standards, and reporting criteria. Because outbreaks are more likely to produce peer-reviewed reports than silent transmission periods, publication bias may exist. Furthermore, limited access to molecular confirmation and inconsistent use of standardised case definitions may have led to under- or overestimation of CHIKV incidence.

Despite notable progress in documenting the epidemiology of CHIKV across Southern Africa, substantial gaps persist that constrain regional preparedness and understanding of the virus’s transmission ecology. Most studies remain concentrated in Madagascar, Mozambique, and the DRC, with only sporadic investigations in Malawi, Angola, Tanzania, and Mauritius. This geographic bias limits a coherent understanding of subregional heterogeneity and cross-border transmission dynamics. For instance, studies in Madagascar revealed sustained co-circulation of CHIKV with dengue and West Nile viruses between 2011 and 2013 (Table 3), yet comparable multi-virus surveillance is absent in several neighboring countries. Similarly, evidence from DRC and Angola demonstrated outbreaks dominated by the ECSA lineage, but genomic data remain too sparse to trace introductions or adaptive mutations across borders (Table 3). A major limitation across the Southern African literature is the heavy reliance on serological assays, often without molecular confirmation. The frequent use of IgM/IgG ELISA in febrile cohorts, though cost-effective raises concerns of cross-reactivity with other alphaviruses and flaviviruses, as seen in Mozambique and Madagascar. Only a handful of studies employed RT-PCR or sequencing, such as Selhorst et al. (2020) in the DRC, which confirmed the ECSA lineage from both human and mosquito samples. This diagnostic inconsistency impedes inter-study comparability and precludes robust meta-analysis of prevalence data. Furthermore, few studies integrated entomological surveillance alongside clinical testing; where such data exist, vector diversity and abundance are poorly correlated with human case distribution, as highlighted by Guarido et al. [18], who found no CHIKV-positive mosquito pools in South Africa despite prior outbreaks.

Another gap lies in population representation and study design. Most investigations are cross-sectional and hospital-based, focusing on acute febrile patients or specific subgroups such as pregnant women or PLWHIV. This narrow sampling excludes asymptomatic or subclinical infections, which can significantly influence herd immunity and transmission persistence. Longitudinal follow-ups are rare; only the Mauritian cohort by Essackjee et al. [10] tracked post-infection outcomes, revealing persistent arthralgia and rheumatoid-like symptoms two years after infection. Such chronic sequelae are likely under-reported elsewhere, leaving the long-term disease burden in Southern Africa largely undefined. Socioeconomic and environmental variables, though occasionally documented, remain underexplored as predictive risk factors. While studies occasionally noted rural–urban contrasts or income-level distribution, few employed spatial or climate-linked modeling. Yet, patterns observed in Tanzania and Mozambique suggest that rainfall, flooding (e.g., post-Cyclone Idai), and unplanned urbanization foster *Aedes* breeding sites, amplifying outbreak potential. Integrating such environmental datasets with epidemiological surveillance would improve forecasting under climate variability scenarios. From a regional health systems perspective, surveillance capacity remains fragmented. Countries like South Africa maintain sophisticated arbovirus networks, but in most SADC nations, case detection depends on externally supported outbreak investigations. This reactive rather than proactive posture delays containment and weakens cross-border information sharing. The absence of harmonized diagnostic algorithms and reference laboratories further limits early detection of emergent lineages.

Future research should prioritize standardized, multi-country surveillance frameworks encompassing both molecular and serological diagnostics. Establishing sentinel sites in high-risk agro-ecological zones could link entomological indices with human seroprevalence. Expanding genomic sequencing coverage across countries such as Malawi, Mozambique, and Angola will clarify lineage evolution and vector adaptation patterns. Longitudinal cohort studies are also essential to quantify chronic outcomes and quality-of-life impacts post-infection, building upon findings from Mauritius. Moreover, integrating climate and land-use modeling with Aedes ecology can yield predictive tools for outbreak risk mapping, a critical step under accelerating climate change. Essentially, the region would benefit from operational research on integrated vector management and community-based surveillance suited to both rural and peri-urban settings. Exploring the cost-effectiveness of novel diagnostic approaches, such as dried blood spot sampling validated in Madagascar, could enhance surveillance reach in resource-limited areas. Strengthening laboratory networks and data sharing under SADC’s “One Health” and cross-border disease surveillance frameworks will be indispensable for anticipating future chikungunya emergence in Southern Africa.

## 6. Conclusion

This comprehensive analysis indicates that CHIKV is geographically prevalent throughout the SADC region, with evidence of recurrent outbreaks and sustained inter-epidemic transmission. The included studies revealed substantial heterogeneity in seroprevalence, clinical presentation, diagnostic approaches, and surveillance capacity across countries and populations. Co-circulation with other arboviruses, coupled with limited molecular diagnostic infrastructure and fragmented surveillance systems, continues to complicate accurate detection and outbreak response. The results emphasize the role of environmental change, urbanization, climate variability, and vector proliferation or expansion in the persistence and dissemination of CHIKV in the region. Although most reported infections were associated with acute febrile illness and arthralgia, several studies also documented severe neurological manifestations and long-term musculoskeletal sequelae, underscoring the broader public health burden of the disease. Therefore, enhancing or strengthening integrated surveillance systems that integrate molecular diagnostics, seroepidemiology, entomological monitoring, and climate-informed risk assessment is crucial for augmenting outbreak preparedness and early detection. Moreover, standardized regional surveillance systems, expanded genomic monitoring, and longitudinal cohort studies are essential for a more accurate characterization of transmission patterns, chronic diseases burden, and population-level risk factors. Collectively, these measures will be critical for reducing future epidemic risk and strengthening public health resilience against CHIKV and other emerging arboviral diseases across Southern Africa.

## Supporting information

S1 File PRISMA Checklist. (https://creativecommons.org/licenses/by/4.0/).
(DOCX)
