# Supplementary material for: Epidemiology, seroprevalence, and circulation of Chikungunya virus in Southern Africa (SADC region): A systematic review
Source: PLoS Negl Trop Dis. 2026 Jun 16;20(6):e0014438. doi: 10.1371/journal.pntd.0014438 (PMC13293513; doi:10.1371/journal.pntd.0014438)
Supplement: Attachment — Submitted filename: Response to Reviewers.docx [file pntd.0014438.s002.docx]

Reviewer tabulated response

| Reviewer`s comments | Activity |
| --- | --- |
| This systematic review's topic is relevant and important for the PLOS Neglected Tropical Diseases audience, especially considering the rising burden of CHIKV in Southern Africa and the need for better surveillance and regional data synthesis. The manuscript addresses a significant gap. However, major revisions are necessary before it can be considered for publication. My primary and minor comments are listed below. | We appreciate the Reviewer's detailed and critical appraisal of our manuscript. We appreciate the awareness of the topic's relevance and the importance of CHIKV data synthesis in Southern Africa. We have addressed all major and small suggestions and significantly updated the paper to increase methodological clarity, consistency, and overall presentation. Our detailed responses are given below. |
| Major Comments  1. Inconsistencies in the number of included studies  The manuscript refers to 20 studies in the Abstract, while the PRISMA flow diagram and text report 22 studies. This must be corrected throughout, and the final number should be consistently reflected in the Abstract, Results, Figure 1, and Table 3. | The authors have corrected the number of included studies to 20 consistently throughout the manuscript, including the Abstract, Results section, PRISMA flow diagram (Figure 1), and Table 3. |
| 2. Inconsistency in time ranges  The Abstract states that studies from 2012–2024 were included, but Table 2 lists “1952–2025”. A clear statement of the exact search time frame and the date of the last search is essential. | The correct search time frame for the systematic review is January 2012 to October 2025, reflecting the period of contemporary CHIKV circulation and surveillance in Southern Africa. The “1952–2025” entry was an error and has been removed. |
| 3. Title wording  The term “Incident” is incorrect in an epidemiological context. It should be “Incidence”. A more precise and more accurate title would be:  “Incidence and epidemiology of Chikungunya virus in Southern Africa: A systematic review.” | Revised the title to:  “Incidence and epidemiology of Chikungunya virus in Southern Africa: A systematic review.” |
| 4. PRISMA compliance and methodological transparency  While PRISMA is mentioned, several essential elements are missing:  o No indication whether a protocol was registered (e.g., PROSPERO).  o The screening procedure (number of reviewers, resolution of discrepancies) is not described.  o Language restrictions (English-only) should be stated and acknowledged as a limitation | We thank the reviewer for highlighting these important elements. The Methods section has been revised to improve PRISMA compliance and transparency.  The study is registered with PROSPERO: CRD420251250401  the protocol can be found online <https://www.crd.york.ac.uk/PROSPERO/view/CRD420251250401>  Added a detailed description of the two-stage screening process, including the number of reviewers involved, independent screening, and resolution of discrepancies through consensus or third-reviewer adjudication.  Clarified that only English-language studies were included and acknowledged this as a limitation in the Discussion. |
| 5. Risk of bias assessment  Although the JBI checklist is mentioned, no table or summary of risk-of-bias results is presented. A dedicated figure or table summarizing the quality assessment of included studies is required. | A dedicated summary of the risk-of-bias assessment has now been included.  The authors added a summary bar chart showing the proportion of studies classified as low, moderate, and high risk of bias (low = 15; moderate = 3; high = 2).  Updated the Methods and Results sections to describe scoring thresholds and interpretation. |
| 6. Data extraction clarity  The Methods section lists the extraction of socio-economic and demographic data that are not consistently available in primary studies. The authors should clarify how missing or inconsistent data were handled. | The Data Extraction subsection has been revised to clarify that socio-economic and demographic variables were extracted only when explicitly reported in the original studies. Missing or inconsistently reported data were recorded as “not reported” and were not imputed.  The authors have also clarified procedures for handling missing or incomplete data in the Methods section. |
| 7. Interpretation and use of Figures 2 and 4  The donut chart and heat map appear semi-quantitative and risk implying a level of precision that is not supported by the data. If retained, they must be clearly described as illustrative only, and their limitations emphasized. Alternatively, consider removing them. | The authors agree with this concern. The purpose and limitations of these figures have been clarified.  The authors have revised figure legends to explicitly state that Figures 2 and 4 are illustrative and descriptive, not quantitative estimates and added explanatory text in the Results section highlighting their semi-quantitative nature and limitations. |
| 8. Scope of One Health / SDG discussion  While valuable, this section is much longer than expected in a systematic review and includes conceptual commentary not directly based on the synthesized evidence. Consider tightening this section and linking it more clearly to the review’s findings. | The authors have reduced the length of the section and removed purely conceptual material. |
| Minor Comments  1. Language and grammar  Numerous grammatical errors and typographical mistakes (e.g., “tittle”, “illegible” instead of “ineligible”) are present throughout the manuscript, along with inconsistent abbreviations (CHKV vs CHIKV). A thorough English-language edit by a fluent or professional editor is highly recommended. | The manuscript has undergone a thorough English-language revision to correct grammatical errors, typographical mistakes, and inconsistent terminology. |
| 2. Consistency of abbreviations  All abbreviations (e.g., SADC, PLWHIV, ECSA) should be defined at first use and used consistently thereafter. | All abbreviations are now defined at first use and applied consistently thereafter. |
| 3. Tables and figures  Ensure that all tables and figures are numbered correctly, cited in the text, and contain self-explanatory legends. | All tables and figures have been checked for numbering, citation, and clarity. |
| Overall, the manuscript addresses an important topic but requires substantive methodological and editorial refinement before proceeding. | We sincerely appreciate the Reviewer's comprehensive and insightful remarks. The text has been significantly updated in response to all criticisms, resulting in greater methodological rigor, transparency, and clarity. We feel the updated version is now in line with the standards and expectations of PLOS Neglected Tropical Diseases. |
|  |  |
