# Supplementary material for: Epidemiology, seroprevalence, and circulation of Chikungunya virus in Southern Africa (SADC region): A systematic review
Source: PLoS Negl Trop Dis. 2026 Jun 16;20(6):e0014438. doi: 10.1371/journal.pntd.0014438 (PMC13293513; doi:10.1371/journal.pntd.0014438)
Supplement: Attachment — Submitted filename: Response_to_Reviewers_auresp_1.docx [file pntd.0014438.s003.docx]

Reviewer #1:

| Reviewer`s comments | Activity |
| --- | --- |
| This manuscript provides a valuable synthesis of evidence on CHIKV in the Southern Africa/SADC region and addresses an important gap in the literature. The study design is appropriate, and the authors indicate adherence to PRISMA with PROSPERO registration. However, several methodological issues require clarification to ensure transparency and reproducibility: | We appreciate the reviewer for this insightful comment. |
| - Geographic scope: The manuscript uses "Southern Africa" and "SADC" interchangeably, while the included countries appear to reflect SADC membership, including countries not consistently classified as "Southern Africa" in geographic terms. Please define the geographic scope explicitly in the Methods, justify the chosen framing, and align the title and terminology accordingly. | We thank the reviewer for this important observation. We acknowledge that the terms “Southern Africa” and “SADC” were previously used interchangeably, which may lead to ambiguity. In this review, our intention was to focus on countries within the Southern African Development Community (SADC), as this regional bloc provides a relevant epidemiological and public health framework for understanding chikungunya virus (CHIKV) transmission, surveillance, and control.  To ensure consistency, we have revised the manuscript to use “SADC region” as the primary descriptor, while retaining “Southern Africa” only where used in a broader contextual sense. The title has also been aligned accordingly to accurately reflect the geographic scope. |
| - Time window: The selected review period (January 2012–October 2025) seems reasonable; please add one sentence explaining the rationale for this start and end dates. | The review period (January 2012 to October 2025) was selected to capture contemporary evidence on chikungunya virus epidemiology in the SADC region following the major global expansion of CHIKV between 2004 and 2011, while ensuring inclusion of the most recent studies reflecting evolving transmission dynamics, improved diagnostic capacity, and the increasing influence of climate and environmental change on arboviral spread. |
| - Language restriction: Limiting the search to English-language publications should be acknowledged clearly as a limitation. | We appreciate the reviewer bringing this crucial matter to our attention. We admit that limiting the search to English-language publications might have resulted in the omission of pertinent studies written in other languages, especially from Francophone and Lusophone countries in the SADC region. This could reduce the evidence base's completeness and add linguistic bias. In the Methods and Discussion sections, we have now made this clear as a restriction. |
| -No ethical concerns are identified | Confirmed |
| **Results:**  The results are generally well-described and cover a broad range of evidence including geographic distribution, seroprevalence, clinical outcomes, and diagnostic approaches. However, several issues require attention: | The authors thank the reviewer for the positive assessment of the Results section and for recognizing the breadth of evidence synthesized in this review. |
| - Results vs. Discussion boundary: The Results section contains substantial interpretive commentary that would be more appropriate in the Discussion. Phrases such as “these patterns suggest,” “this breadth demonstrates,” and recommendations for integrated surveillance appear throughout the Results and should be moved to the Discussion. The Results should report findings only. | The authors have carefully revised the Results section to remove interpretive and inferential language (e.g., phrases such as “these patterns suggest,” “this breadth demonstrates,” and forward-looking recommendations). The Results section now strictly presents descriptive findings derived from the included studies. |
| - Incidence: The title and a Results subheading reference “incidence,” but no actual incidence measures are presented. The evidence summarized appears to be seroprevalence/seropositivity estimates and outbreak case counts. The authors should replace “incidence” with a more appropriate term in the title and subheadings (e.g., “circulation,” “seroprevalence,” or “occurrence/burden”), or a term of their choosing, or clearly define and report incidence measures if they were extracted. | Epidemiology, seroprevalence, and circulation of Chikungunya virus in Southern Africa (SADC region): A systematic review |
| -Geographic error: The Results section lists Cabo Delgado and Sofala provinces as DRC outbreak locations. These are provinces in Mozambique, not the DRC. Please correct. | Corrected |
| -Figure 3: The chart uses overlapping categories (pregnant women are a subset of adult females; PLWHIV span multiple age/sex groups), yet proportions sum to 100%, risking misinterpretation. Please state the denominator and classification rules explicitly (participants vs. studies and how overlaps were handled) or redesign with mutually exclusive categories. The caveat that proportions reflect study sampling/research focus rather than disease distribution should appear in the figure caption rather than only in the body text. | Figure 3. Representation of study populations across included chikungunya virus (CHIKV) studies in the Southern African Development Community. |
| -Figure 4: Please clarify in the caption what the y-axis represents and whether segments reflect number of studies, reports, or simple presence/absence of each virus per country. | The y-axis represents the included countries, while the x-axis displays the arboviruses reported in each setting. Colored segments indicate the presence of each virus as documented in at least one included study per country. The figure reflects reported occurrence only (presence/absence) and does not represent the number of cases or the frequency of detection. Multiple viruses within a country indicate co-reporting in the literature rather than simultaneous co-infection within individuals. |
| -Figure 5: The heat map visually implies comparability across countries and populations despite heterogeneity in diagnostic assays, thresholds, and study designs. Please make the source/derivation of each cell transparent and specify the rule used to select values when multiple estimates were available (including sample size, assay type, and whether IgM vs IgG). Angola is shown with a high estimate (47% among febrile patients) derived from a relatively small clinical cohort (n=57 suspected cases); this context should be clearly indicated to avoid over-interpretation relative to population-based estimates from other settings. Please avoid the term “intensity,” clearly mark cells with no data, and provide a supplementary table mapping each value to its source study (or consider replacing the heat map with a study-level table or range-based summary). | Figure 5, presented as an illustrative summary of reported seroprevalence patterns and should not be interpreted as a standardized comparison across countries or populations |
| -Section numbering: Results subsections are numbered 2.5 through 2.8 rather than 3.1 through 3.4. Please correct throughout. | Corrected |
| **Conclusion:**  The study is a valuable contribution and the conclusions are supported by the data presented. The public health relevance is clearly articulated, and the limitations are appropriately acknowledged. Addressing the points raised above will further strengthen the manuscript prior to publication. | The authors have undertaken substantial revisions to strengthen methodological transparency, clarity, and overall rigor of the manuscript. |
|  |  |

Reviewer #2:

| Reviewer’s comments | Activity |
| --- | --- |
| The manuscript “Incidence and epidemiology of Chikungunya virus in Southern Africa: A systematic review” has been previously reviewed and revised based on reviewer suggestions. The study aims to document prevalence and status of CHIK in SADC through literature published between 2012-2025, providing insights into the epidemiology, disease burden, and consequences in the SADC region caused by CHIKV. The review also examines socio-economic factors influencing CHIKV transmission and management, as well the role of geographical variation in shaping virus transmission and maintenance. The study addresses four key questions:  1) What is the reported incidence and geographic distribution of CHIKV in Southern Africa?  2) Which mosquito vectors are most frequently implicated in transmission?  3) How do viral genotypes vary across outbreaks and how are they linked to severity?  4) Which populations are most affected, and what are the documented clinical outcomes and fatalities?  The methodology used to conduct this systematic review is appropriate and followed PRISMA guidelines and registered with PROPERO | The authors deeply appreciate the reviewer's positive assessment of our work and the recognition of the systematic review's importance in addressing the epidemiology and public health implications of the chikungunya virus (CHIKV) in the Southern African Development Community (SADC) region.  The authors have further strengthened the manuscript by improving methodological transparency, refining the Results and Discussion sections to ensure strict PRISMA-compliant reporting, clarifying geographic scope and terminology related to the SADC region, and revising figures and tables to avoid overinterpretation of heterogeneous data. We also revised the title and terminology to better reflect the nature of the synthesized evidence and corrected inconsistencies identified throughout the manuscript. |
| **Results:**  -The study performed for Reference #10 was actually conducted in 2008, even though it was published in 2013, so would this be relevant for this review, considering that the actual study was performed outside of the timeframe? Additionally, Reference #16 was published in 2013, but the actual study took place in 2010. The authors need to make clear the distinction between study time frame vs publication time frame - the authors need to clarify that a publication time frame was used as some publications used data sets from 2008-2010, etc. | In this systematic review, the eligibility criterion was based on the publication period (January 2012–October 2025) rather than the dates of field data collection. As a result, some of the included studies that were published during this period reported data collected prior to 2012, such as Reference [10], which examined results from the 2005–2006 Mauritius outbreak and was published in 2013, and Reference [16], which reported data collected during the 2010 Madagascar outbreak but was also published in 2013. These studies were kept because they offered pertinent clinical, diagnostic, and epidemiological data that was released throughout the predetermined review period.  To address this concern, we have clarified the distinction between publication timeframe and study data collection period in the methods section and have revised the results section where appropriate. |
| -Figure 5 legend makes it seem like seroprevalence was determined for years 2012-2025. The legend time frame should specify if this is a publication time frame (2012-2025) or study time frame (2008-2025). | Figure 5. Study-level CHIKV seroprevalence findings reported across Southern African Development Community (SADC) countries in studies published between January 2012 and October 2025. |
| -Figure 4: It is difficult to determine co-circulation as most of the studies presented are biased towards chikungunya virus. The authors would have to include studies that looked at other arboviruses during the 2012-2025 time-frame. Figure 4 and mention of co-circulation on pages 14 and 15 should be removed entirely unless a more in-depth analysis is performed. The inaccuracy to portray co-circulation can be presented in the discussion, due to limitations of studies and bias towards chikungunya. | The authors have included a more in-depth analysis in accordance with the reviewer`s comments. |
| -Page 15: Statement that Malawi, South Africa, and Mauritius reported chikungunya as the sole arbovirus detected is biased and misleading. Reference #10 only based chikungunya on clinically diagnosed chikungunya infection based on patient symptoms, which was not confirmed with laboratory testing. This study did not investigate other circulating arboviral infections. Reference #18 only used alphavirus genus-specific nested RT-PCR and did not investigate other co-circulating arboviruses such as RVFV, which was reported in within the same time frame as Reference #18 (https://doi.org/10.3389/fvets.2021.730424). | To avoid overinterpretation and potential bias, we have removed the statement that these countries “reported chikungunya as the sole arbovirus detected.” The revised text now focuses strictly on the findings reported within the included CHIKV-focused studies and avoids broader inferences regarding absence of other arboviruses. |
| -Page 16: Authors mention that Mauritius reported the highest post-outbreak antibody persistence (>50%) two years after infection, yet no serological studies were performed whatsoever in the referenced study. The referenced study merely selected participants who submitted self-reported questionnaires if they experienced musculoskeletal symptoms which were preceded by any one of fever, arthralgia and rash. The referenced study is biased as it lacks laboratory confirmation for ailments that could have been caused by other circulating infectious disease or underlying conditions and this reference should be removed from the analysis in the systematic review. | The authors have removed all references to “antibody persistence” and revised the corresponding Results and Discussion sections to accurately reflect the methodology and limitations of the study. We also acknowledge the potential for recall bias, symptom misclassification, and confounding by other infectious or chronic musculoskeletal conditions due to the absence of laboratory confirmation during long-term follow-up. However, we respectfully retained the study in the review because it still provides relevant observational evidence regarding reported long-term musculoskeletal outcomes following the documented chikungunya outbreak in Mauritius. |
| -Page 17: mention of reference #10 where patients were followed for more than two years after infection, which is false. Participants weren’t followed for two years after the outbreak as they were actively recruited from health and village centres two years after the CHIK outbreak and self-reported their symptoms on questionnaires. Based on their reply on the questionnaire, this was clinically diagnosed as CHIK without laboratory confirmation if it met two out of three criteria for fever, arthralgia and rash – considering that DENV and malaria are also circulating in this region, people self-reporting these symptoms could have been infected with other infectious diseases or have other underlying conditions. Additionally, respondents to the study were predominantly female, but the authors attributed this to their sampling methods and the fact that females were easier to recruit as they were more present at health and village centres during working hours, which created bias for reporting that females were predominantly infected with CHIK. Although clinical diagnosis can be acceptable for systematic review, I believe this reference should be removed entirely given the amount of bias that can be introduced from this one study based on self-reporting of symptoms and the fact that DENV and malaria is rampant in this region and without diagnostic confirmation, symptoms can be mistaken for CHIK | We agree that the previous wording overstated the strength of evidence provided by the Mauritian study and may have implied longitudinal laboratory-confirmed follow-up, which was not the case. The authors have carefully reassessed the study against our eligibility criteria. Because the review included observational studies reporting clinically suspected CHIKV-associated outcomes during documented outbreaks, we retained the study but substantially downgraded the interpretation and removed all causal or confirmatory language implying laboratory-verified chronic CHIKV morbidity. |
| **Conclusion:**  The conclusions in the manuscript are mainly supported data presented and limitations are discussed. However, as previously mentioned, reference # 10 is biased (Essackjee et al) and should be removed from the analysis. These changes will need to be addressed in the text and all the figures and tables presented in this manuscript. Other than that, this is the first systematic review performed for CHIKV in the SADC regions which is addresses gaps in surveillance and diagnostics for this pathogen. | We acknowledge the reviewer’s thorough assessment of Reference #10 (Essackjee et al.) and concur that the study possesses significant methodological shortcomings, notably the lack of laboratory validation, retroactive recruiting, potential recall bias, and probable gender-selection bias. We also acknowledge that the study should not be interpreted as evidence of seroprevalence, antibody persistence, or laboratory-confirmed incidence. In response, we have comprehensively revised the manuscript to ensure that the findings from this study are represented appropriately and cautiously.  The authors have elected not to remove the study entirely because it remains one of the few investigations from the Southern African region that specifically examined long-term musculoskeletal outcomes following a major chikungunya outbreak. Although the study relied on clinically defined CHIKV cases identified during a recognized epidemic context, the authors explicitly acknowledged these diagnostic limitations and justified the use of syndromic case definitions based on the classical symptom triad (fever, arthralgia, and rash) during a documented outbreak setting. Such clinically based outbreak definitions have historically been used in arboviral epidemiology, particularly in resource-limited settings where confirmatory diagnostics were restricted. |
| -Major: Removal of reference #10 will require authors to reanalyze their data sets. This can receive mention in their discussion but this study would not be appropriate for their conclusions. This reference is used multiple times throughout the manuscript and its use needs to be reevaluated or removed completely. Example: this reference was used in Figure 5 for seroprevalence for Mauritius, but this study never determined seroprevalence. | The authors have elected not to remove the study entirely because it remains one of the few investigations from the Southern African region that specifically examined long-term musculoskeletal outcomes following a major chikungunya outbreak. Although the study relied on clinically defined CHIKV cases identified during a recognized epidemic context, the authors explicitly acknowledged these diagnostic limitations and justified the use of syndromic case definitions based on the classical symptom triad (fever, arthralgia, and rash) during a documented outbreak setting. Such clinically based outbreak definitions have historically been used in arboviral epidemiology, particularly in resource-limited settings where confirmatory diagnostics were restricted.  Furthermore, the primary relevance of Essackjee et al. to this systematic review is not seroprevalence estimation, incidence quantification, or molecular epidemiology, but rather its contribution to understanding the potential chronic rheumatologic burden associated with suspected CHIKV infection in Southern Africa. The study provides region-specific evidence suggesting prolonged musculoskeletal morbidity following outbreak-associated illness, which aligns with observations from laboratory-confirmed cohorts reported elsewhere in Réunion Island, France, and other CHIKV-endemic settings. Consequently, retaining the study contributes contextual value to the review’s broader objective of synthesizing the epidemiological and clinical consequences of CHIKV in the SADC region.  To address the reviewer’s concerns and avoid overinterpretation, we implemented the following revisions throughout the manuscript:   - Removed all references to “seroprevalence,” “antibody persistence,” and “laboratory-confirmed infection” associated with the Mauritius study. - Removed Mauritius-derived values from Figure 5 and any quantitative prevalence summaries. - Revised all Results and Discussion sections to clearly state that the Mauritius study reported self-reported chronic musculoskeletal symptoms following clinically suspected CHIKV infection during a documented outbreak. - Added explicit caveats regarding the lack of laboratory confirmation, possible misclassification with dengue or malaria, retrospective symptom reporting, and recruitment bias. - Avoided using the study to support conclusions regarding population-level burden, transmission intensity, or demographic risk distribution. - Restricted interpretation of the study to exploratory evidence of possible long-term post-CHIKV musculoskeletal sequelae.   The revised text now reads more cautiously, for example:  “Post-outbreak investigations in Mauritius described persistent musculoskeletal symptoms among individuals with clinically suspected CHIKV infection identified during the 2006 epidemic; however, these findings should be interpreted cautiously because diagnoses were not laboratory confirmed and relied on retrospective self-reported symptom histories [10].” |
