# Supplementary material for: Epidemiology, seroprevalence, and circulation of Chikungunya virus in Southern Africa (SADC region): A systematic review
Source: PLoS Negl Trop Dis. 2026 Jun 16;20(6):e0014438. doi: 10.1371/journal.pntd.0014438 (PMC13293513; doi:10.1371/journal.pntd.0014438)
Supplement: Peer review history

# Author comment: Author response to Decision Letter 0

**DOI:** 10.1371/journal.pntd.0014438.r001

14 Jan 2026

Attachment Submitted filename: Response to Reviewers.docx

# Peer review: Decision Letter 0

**DOI:** 10.1371/journal.pntd.0014438.r002

**Authors:** David Safronetz

27 Mar 2026

PNTD-D-26-00088

Incidence and epidemiology of Chikungunya virus in Southern Africa: A systematic review

PLOS Neglected Tropical Diseases

Dear Dr. Lamula,

Thank you for submitting your manuscript to PLOS Neglected Tropical Diseases. After careful consideration, we feel that it has merit but does not fully meet PLOS Neglected Tropical Diseases's publication criteria as it currently stands. Therefore, we invite you to submit a revised version of the manuscript that addresses the points raised during the review process.

Please submit your revised manuscript within by May 26 2026 11:59PM. If you will need more time than this to complete your revisions, please reply to this message or contact the journal office at plosntds@plos.org. When you're ready to submit your revision, log on to https://www.editorialmanager.com/pntd/ and select the 'Submissions Needing Revision' folder to locate your manuscript file.

Please include the following items when submitting your revised manuscript:

* A letter that responds to each point raised by the editor and reviewer(s). You should upload this letter as a separate file labeled 'Response to Reviewers'. This file does not need to include responses to any formatting updates and technical items listed in the 'Journal Requirements' section below.

* A marked-up copy of your manuscript that highlights changes made to the original version. You should upload this as a separate file labeled 'Revised Manuscript with Track Changes'.

* An unmarked version of your revised paper without tracked changes. You should upload this as a separate file labeled 'Manuscript'.

If you would like to make changes to your financial disclosure, competing interests statement, or data availability statement, please make these updates within the submission form at the time of resubmission. Guidelines for resubmitting your figure files are available below the reviewer comments at the end of this letter.

We look forward to receiving your revised manuscript.

Kind regards,

David Safronetz, Ph.D.

Section Editor

PLOS Neglected Tropical Diseases

David Safronetz

Section Editor

PLOS Neglected Tropical Diseases

Shaden Kamhawi

co-Editor-in-Chief

PLOS Neglected Tropical Diseases

orcid.org/0000-0003-4304-636XX

Paul Brindley

co-Editor-in-Chief

PLOS Neglected Tropical Diseases

orcid.org/0000-0003-1765-0002

**Journal Requirements:**

1) Please ensure that the CRediT author contributions listed for every co-author are completed accurately and in full.

At this stage, the following Authors/Authors require contributions: Siphamandla Lamula, Ndivhuwo Ramatsitsi, and Lisa Buwa-Komoreng. Please ensure that the full contributions of each author are acknowledged in the "Add/Edit/Remove Authors" section of our submission form.

The list of CRediT author contributions may be found here: https://journals.plos.org/plosntds/s/authorship#loc-author-contributions

2) Please provide an Author Summary. This should appear in your manuscript between the Abstract (if applicable) and the Introduction, and should be 150-200 words long. The aim should be to make your findings accessible to a wide audience that includes both scientists and non-scientists. Sample summaries can be found on our website under Submission Guidelines:

https://journals.plos.org/plosntds/s/submission-guidelines#loc-parts-of-a-submission

3) Please upload all main figures as separate Figure files in .tif or .eps format. For more information about how to convert and format your figure files please see our guidelines:

https://journals.plos.org/plosntds/s/figures

**Reviewers' Comments:**

Reviewer's Responses to Questions

**Key Review Criteria Required for Acceptance?**

As you describe the new analyses required for acceptance, please consider the following:

**Methods**

-Are the objectives of the study clearly articulated with a clear testable hypothesis stated?

-Is the study design appropriate to address the stated objectives?

-Is the population clearly described and appropriate for the hypothesis being tested?

-Is the sample size sufficient to ensure adequate power to address the hypothesis being tested?

-Were correct statistical analysis used to support conclusions?

-Are there concerns about ethical or regulatory requirements being met?

Reviewer #1: This manuscript provides a valuable synthesis of evidence on CHIKV in the Southern Africa/SADC region and addresses an important gap in the literature. The study design is appropriate, and the authors indicate adherence to PRISMA with PROSPERO registration. However, several methodological issues require clarification to ensure transparency and reproducibility:

- Geographic scope: The manuscript uses "Southern Africa" and "SADC" interchangeably, while the included countries appear to reflect SADC membership, including countries not consistently classified as "Southern Africa" in geographic terms. Please define the geographic scope explicitly in the Methods, justify the chosen framing, and align the title and terminology accordingly.

- Time window: The selected review period (January 2012–October 2025) seems reasonable; please add one sentence explaining the rationale for this start and end dates.

- Language restriction: Limiting the search to English-language publications should be acknowledged clearly as a limitation.

-No ethical concerns are identified

Reviewer #2: The manuscript “Incidence and epidemiology of Chikungunya virus in Southern Africa: A systematic review” has been previously reviewed and revised based on reviewer suggestions. The study aims to document prevalence and status of CHIK in SADC through literature published between 2012-2025, providing insights into the epidemiology, disease burden, and consequences in the SADC region caused by CHIKV. The review also examines socio-economic factors influencing CHIKV transmission and management, as well the role of geographical variation in shaping virus transmission and maintenance. The study addresses four key questions:

1) What is the reported incidence and geographic distribution of CHIKV in Southern Africa?

2) Which mosquito vectors are most frequently implicated in transmission?

3) How do viral genotypes vary across outbreaks and how are they linked to severity?

4) Which populations are most affected, and what are the documented clinical outcomes and fatalities?

The methodology used to conduct this systematic review is appropriate and followed PRISMA guidelines and registered with PROPERO.

**********

**Results**

-Does the analysis presented match the analysis plan?

-Are the results clearly and completely presented?

-Are the figures (Tables, Images) of sufficient quality for clarity?

Reviewer #1: The results are generally well-described and cover a broad range of evidence including geographic distribution, seroprevalence, clinical outcomes, and diagnostic approaches. However, several issues require attention:

- Results vs. Discussion boundary: The Results section contains substantial interpretive commentary that would be more appropriate in the Discussion. Phrases such as “these patterns suggest,” “this breadth demonstrates,” and recommendations for integrated surveillance appear throughout the Results and should be moved to the Discussion. The Results should report findings only.

- Incidence: The title and a Results subheading reference “incidence,” but no actual incidence measures are presented. The evidence summarized appears to be seroprevalence/seropositivity estimates and outbreak case counts. The authors should replace “incidence” with a more appropriate term in the title and subheadings (e.g., “circulation,” “seroprevalence,” or “occurrence/burden”), or a term of their choosing, or clearly define and report incidence measures if they were extracted.

Geographic error: The Results section lists Cabo Delgado and Sofala provinces as DRC outbreak locations. These are provinces in Mozambique, not the DRC. Please correct.

Figure 3: The chart uses overlapping categories (pregnant women are a subset of adult females; PLWHIV span multiple age/sex groups), yet proportions sum to 100%, risking misinterpretation. Please state the denominator and classification rules explicitly (participants vs. studies and how overlaps were handled) or redesign with mutually exclusive categories. The caveat that proportions reflect study sampling/research focus rather than disease distribution should appear in the figure caption rather than only in the body text.

Figure 4: Please clarify in the caption what the y-axis represents and whether segments reflect number of studies, reports, or simple presence/absence of each virus per country.

Figure 5: The heat map visually implies comparability across countries and populations despite heterogeneity in diagnostic assays, thresholds, and study designs. Please make the source/derivation of each cell transparent and specify the rule used to select values when multiple estimates were available (including sample size, assay type, and whether IgM vs IgG). Angola is shown with a high estimate (47% among febrile patients) derived from a relatively small clinical cohort (n=57 suspected cases); this context should be clearly indicated to avoid over-interpretation relative to population-based estimates from other settings. Please avoid the term “intensity,” clearly mark cells with no data, and provide a supplementary table mapping each value to its source study (or consider replacing the heat map with a study-level table or range-based summary).

Section numbering: Results subsections are numbered 2.5 through 2.8 rather than 3.1 through 3.4. Please correct throughout.

Reviewer #2: The study performed for Reference #10 was actually conducted in 2008, even though it was published in 2013, so would this be relevant for this review, considering that the actual study was performed outside of the timeframe? Additionally, Reference #16 was published in 2013, but the actual study took place in 2010. The authors need to make clear the distinction between study time frame vs publication time frame - the authors need to clarify that a publication time frame was used as some publications used data sets from 2008-2010, etc.

Figure 5 legend makes it seem like seroprevalence was determined for years 2012-2025. The legend time frame should specify if this is a publication time frame (2012-2025) or study time frame (2008-2025).

Figure 4: It is difficult to determine co-circulation as most of the studies presented are biased towards chikungunya virus. The authors would have to include studies that looked at other arboviruses during the 2012-2025 time-frame. Figure 4 and mention of co-circulation on pages 14 and 15 should be removed entirely unless a more in-depth analysis is performed. The inaccuracy to portray co-circulation can be presented in the discussion, due to limitations of studies and bias towards chikungunya.

Page 15: Statement that Malawi, South Africa, and Mauritius reported chikungunya as the sole arbovirus detected is biased and misleading. Reference #10 only based chikungunya on clinically diagnosed chikungunya infection based on patient symptoms, which was not confirmed with laboratory testing. This study did not investigate other circulating arboviral infections. Reference #18 only used alphavirus genus-specific nested RT-PCR and did not investigate other co-circulating arboviruses such as RVFV, which was reported in within the same time frame as Reference #18 (https://doi.org/10.3389/fvets.2021.730424).

Page 16: Authors mention that Mauritius reported the highest post-outbreak antibody persistence (>50%) two years after infection, yet no serological studies were performed whatsoever in the referenced study. The referenced study merely selected participants who submitted self-reported questionnaires if they experienced musculoskeletal symptoms which were preceded by any one of fever, arthralgia and rash. The referenced study is biased as it lacks laboratory confirmation for ailments that could have been caused by other circulating infectious disease or underlying conditions and this reference should be removed from the analysis in the systematic review.

Page 17: mention of reference #10 where patients were followed for more than two years after infection, which is false. Participants weren’t followed for two years after the outbreak as they were actively recruited from health and village centres two years after the CHIK outbreak and self-reported their symptoms on questionnaires. Based on their reply on the questionnaire, this was clinically diagnosed as CHIK without laboratory confirmation if it met two out of three criteria for fever, arthralgia and rash – considering that DENV and malaria are also circulating in this region, people self-reporting these symptoms could have been infected with other infectious diseases or have other underlying conditions. Additionally, respondents to the study were predominantly female, but the authors attributed this to their sampling methods and the fact that females were easier to recruit as they were more present at health and village centres during working hours, which created bias for reporting that females were predominantly infected with CHIK. Although clinical diagnosis can be acceptable for systematic review, I believe this reference should be removed entirely given the amount of bias that can be introduced from this one study based on self-reporting of symptoms and the fact that DENV and malaria is rampant in this region and without diagnostic confirmation, symptoms can be mistaken for CHIK.

**********

**Conclusions**

-Are the conclusions supported by the data presented?

-Are the limitations of analysis clearly described?

-Do the authors discuss how these data can be helpful to advance our understanding of the topic under study?

-Is public health relevance addressed?

Reviewer #1: The study is a valuable contribution and the conclusions are supported by the data presented. The public health relevance is clearly articulated, and the limitations are appropriately acknowledged. Addressing the points raised above will further strengthen the manuscript prior to publication.

Reviewer #2: The conclusions in the manuscript are mainly supported data presented and limitations are discussed. However, as previously mentioned, reference # 10 is biased (Essackjee et al) and should be removed from the analysis. These changes will need to be addressed in the text and all the figures and tables presented in this manuscript. Other than that, this is the first systematic review performed for CHIKV in the SADC regions which is addresses gaps in surveillance and diagnostics for this pathogen.

Major: Removal of reference #10 will require authors to reanalyze their data sets. This can receive mention in their discussion but this study would not be appropriate for their conclusions. This reference is used multiple times throughout the manuscript and its use needs to be reevaluated or removed completely. Example: this reference was used in Figure 5 for seroprevalence for Mauritius, but this study never determined seroprevalence.

**********

**Editorial and Data Presentation Modifications?**

Use this section for editorial suggestions as well as relatively minor modifications of existing data that would enhance clarity. If the only modifications needed are minor and/or editorial, you may wish to recommend “Minor Revision” or “Accept”.

Reviewer #1: Typos and consistency: Please correct minor typographical errors throughout, including "tittle" instead of "title" in section 2.3. Section numbering is inconsistent throughout — results subsections are labeled 2.5 through 2.8 instead of 3.1 through 3.4, and the discussion is labeled Section 3 but subsections are numbered 4.1 through 4.6.

Figure 3: Consider revising to avoid overlapping categories or clarify classification rules in the caption. The percentage numbers appear outside the circle and should be corrected for presentation.

Figure 7 requires careful revision prior to publication as it contains multiple typographical errors including 'Dsangostic amd' instead of 'Diagnostic and', 'cross-recty-nyseny' instead of 'cross-reactivity', and 'vector comprency' instead of 'vector competency', and the figure appears incomplete with text cut off in the lower right panel.

Reviewer #2: Page 4: IXCHIQ vaccine approved in Canada, US and Europe, VIMKUNYA is also approved in the US. The authors need to update the statement on page 4 when they mention that there are no vaccines available for CHIKV.

Page 34: “The frequent use of IgM/IgG ELISA in febrile cohorts, though cost effective raises concerns of cross-reactivity…” – although this statement is true, this can be remedied by paired serology or confirmatory assay such as the PRNT, which the authors should mention. This sentence also needs a reference.

Page 34: reference #10 again used, even though the study didn’t actually track post infection outcomes as cases weren’t confirmed and were merely self reported in a questionnaire.

**********

**Summary and General Comments**

Use this section to provide overall comments, discuss strengths/weaknesses of the study, novelty, significance, general execution and scholarship. You may also include additional comments for the author, including concerns about dual publication, research ethics, or publication ethics. If requesting major revision, please articulate the new experiments that are needed.

Reviewer #1: This manuscript addresses an important and timely topic and provides a valuable synthesis of evidence on CHIKV circulation, clinical presentation, and surveillance across the Southern Africa/SADC region. Strengths include the relevance to neglected tropical disease surveillance, the breadth of included study designs, PRISMA adherence with PROSPERO registration, and a limitations section that is commendably transparent. However several concerns require attention before publication.

The introduction cites reference [11] to support the claim that Culex and Anopheles mosquitoes have been reported to transmit CHIKV in Kenya, Mozambique and South Africa. This claim does not appear to be supported by reference [11] Please provide the correct citation.

The Discussion could be strengthened by reducing repetition and emphasizing synthesis/interpretation. Some subsections (e.g., 4.1–4.2) largely restate Results, and recommendations for integrated surveillance recur across multiple subsections. Consolidating overlapping content and presenting a more thematic synthesis would improve readability and impact

Section 4.6 on One Health, while conceptually relevant, extends beyond the scope of the synthesized evidence and would be more appropriate as supplementary material. The authors are encouraged to briefly acknowledge the One Health framework within existing subsections where directly supported by study findings, rather than maintaining it as a standalone section. Figures 6 and 7 are conceptual rather than data-driven. Figure 7 in particular requires careful revision as it contains multiple typographical errors and appears incomplete with text cut off in the lower right panel.

Overall this manuscript makes a relevant contribution to the literature on arboviral disease in Southern Africa and with the revisions outlined above has the potential to be a valuable resource for researchers and public health practitioners in the region.

Reviewer #2: This systematic review is the first to look at CHIKV circulation in the SADC region. It provides insight into the incidence of CHIKV in the various countries affected and identifies gaps where more testing and surveillance is required.

As mentioned previously, Figure 4 for co-circulating arboviruses is presented poorly as the studies in question are biased towards CHIKV. I believe Figure 4 is inappropriate and irrelevant to this systematic review and should be removed as the review is focused on CHIKV circulation.

**********

PLOS authors have the option to publish the peer review history of their article (what does this mean?). If published, this will include your full peer review and any attached files.

If you choose “no”, your identity will remain anonymous but your review may still be made public.

**Do you want your identity to be public for this peer review?**   For information about this choice, including consent withdrawal, please see our Privacy Policy.

Reviewer #1: No

Reviewer #2: **Yes:**   Alexander Bello

[NOTE: If reviewer comments were submitted as an attachment file, they will be attached to this email and accessible via the submission site. Please log into your account, locate the manuscript record, and check for the action link "View Attachments". If this link does not appear, there are no attachment files.]

**Figure resubmission:**

While revising your submission, we strongly recommend that you use PLOS’s NAAS tool (https://ngplosjournals.pagemajik.ai/artanalysis) to test your figure files. NAAS can convert your figure files to the TIFF file type and meet basic requirements (such as print size, resolution), or provide you with a report on issues that do not meet our requirements and that NAAS cannot fix.

After uploading your figures to PLOS’s NAAS tool - https://ngplosjournals.pagemajik.ai/artanalysis, NAAS will process the files provided and display the results in the "Uploaded Files" section of the page as the processing is complete. If the uploaded figures meet our requirements (or NAAS is able to fix the files to meet our requirements), the figure will be marked as "fixed" above. If NAAS is unable to fix the files, a red "failed" label will appear above. When NAAS has confirmed that the figure files meet our requirements, please download the file via the download option, and include these NAAS processed figure files when submitting your revised manuscript.

**Reproducibility:**

To enhance the reproducibility of your results, we recommend that authors of applicable studies deposit laboratory protocols in protocols.io, where a protocol can be assigned its own identifier (DOI) such that it can be cited independently in the future. Additionally, PLOS ONE offers an option to publish peer-reviewed clinical study protocols. Read more information on sharing protocols at https://plos.org/protocols?utm_medium=editorial-email&utm_source=authorletters&utm_campaign=protocols

# Author comment: Author response to Decision Letter 1

**DOI:** 10.1371/journal.pntd.0014438.r003

25 May 2026

Attachment Submitted filename: Response_to_Reviewers_auresp_1.docx

# Peer review: Decision Letter 1

**DOI:** 10.1371/journal.pntd.0014438.r004

**Authors:** David Safronetz

4 Jun 2026

Dear Dr Lamula,

We are pleased to inform you that your manuscript 'Epidemiology, seroprevalence, and circulation of Chikungunya virus in Southern Africa (SADC region): A systematic review' has been provisionally accepted for publication in PLOS Neglected Tropical Diseases.

Before your manuscript can be formally accepted you will need to complete some formatting changes, which you will receive in a follow up email. A member of our team will be in touch with a set of requests.

Please note that your manuscript will not be scheduled for publication until you have made the required changes, so a swift response is appreciated.

IMPORTANT: The editorial review process is now complete. PLOS will only permit corrections to spelling, formatting or significant scientific errors from this point onwards. Requests for major changes, or any which affect the scientific understanding of your work, will cause delays to the publication date of your manuscript.

Should you, your institution's press office or the journal office choose to press release your paper, you will automatically be opted out of early publication. We ask that you notify us now if you or your institution is planning to press release the article. All press must be co-ordinated with PLOS.

Thank you again for supporting Open Access publishing; we are looking forward to publishing your work in PLOS Neglected Tropical Diseases.

Best regards,

David Safronetz, Ph.D.

Section Editor

PLOS Neglected Tropical Diseases

David Safronetz

Section Editor

PLOS Neglected Tropical Diseases

Shaden Kamhawi

co-Editor-in-Chief

PLOS Neglected Tropical Diseases

orcid.org/0000-0003-4304-636XX

Paul Brindley

co-Editor-in-Chief

PLOS Neglected Tropical Diseases

orcid.org/0000-0003-1765-0002

***********************************************************

p.p1 {margin: 0.0px 0.0px 0.0px 0.0px; line-height: 16.0px; font: 14.0px Arial; color: #323333; -webkit-text-stroke: #323333}span.s1 {font-kerning: none

Reviewer's Responses to Questions

**Key Review Criteria Required for Acceptance?**

As you describe the new analyses required for acceptance, please consider the following:

**Methods**

-Are the objectives of the study clearly articulated with a clear testable hypothesis stated?

-Is the study design appropriate to address the stated objectives?

-Is the population clearly described and appropriate for the hypothesis being tested?

-Is the sample size sufficient to ensure adequate power to address the hypothesis being tested?

-Were correct statistical analysis used to support conclusions?

-Are there concerns about ethical or regulatory requirements being met?

Reviewer #1: The authors have addressed the methodological concerns raised. No further revisions are needed in this section.

Reviewer #2: The authors have addressed all my previous concerns.

**********

**Results**

-Does the analysis presented match the analysis plan?

-Are the results clearly and completely presented?

-Are the figures (Tables, Images) of sufficient quality for clarity?

Reviewer #1: The authors have addressed the results concerns raised. No further revisions are needed in this section.

Reviewer #2: Author responses to my previous concerns are satisfactory.

**********

**Conclusions**

-Are the conclusions supported by the data presented?

-Are the limitations of analysis clearly described?

-Do the authors discuss how these data can be helpful to advance our understanding of the topic under study?

-Is public health relevance addressed?

Reviewer #1: The authors have addressed the results concerns raised. No further revisions are needed in this section

Reviewer #2: Conclusions are satisfactory.

**********

**Editorial and Data Presentation Modifications?**

Use this section for editorial suggestions as well as relatively minor modifications of existing data that would enhance clarity. If the only modifications needed are minor and/or editorial, you may wish to recommend “Minor Revision” or “Accept”.

Reviewer #1: (No Response)

Reviewer #2: I might have forgotten to include this in my previous review but in the introduction on page 5 for the sentence "Currently, there are no licensed antivirals, therapeutic strategies, or vaccines available to remedy CHIKV", this is not true. There are a few licensed CHIKV vaccines such as VIMKUNYA and IXCHIQ, which will need to be added to the text.

**********

**Summary and General Comments**

Use this section to provide overall comments, discuss strengths/weaknesses of the study, novelty, significance, general execution and scholarship. You may also include additional comments for the author, including concerns about dual publication, research ethics, or publication ethics. If requesting major revision, please articulate the new experiments that are needed.

Reviewer #1: I thank the authors for addressing the majority of the concerns raised. One outstanding issue remains — the introduction cites reference [11] to support the claim that Culex and Anopheles mosquitoes have been reported to transmit CHIKV in Kenya, Mozambique and South Africa. This point was not addressed in the authors' response and the correct citation supporting this specific claim should be confirmed and provided before publication.

Reviewer #2: The authors of the manuscript “Incidence and epidemiology of Chikungunya virus in Southern Africa: A systematic review” has addressed all my previous concerns and I am satisfied with their responses. The study is novel and aims to document prevalence and status of CHIK in SADC through literature published between 2012-2025, providing insights into the

epidemiology, disease burden, and consequences in the SADC region caused by CHIKV. The review also examines socio-economic factors influencing CHIKV transmission and management, as well the role of geographical variation in shaping

virus transmission and maintenance.

**********

PLOS authors have the option to publish the peer review history of their article (what does this mean?). If published, this will include your full peer review and any attached files.

If you choose “no”, your identity will remain anonymous but your review may still be made public.

**Do you want your identity to be public for this peer review?**   For information about this choice, including consent withdrawal, please see our Privacy Policy.

Reviewer #1: No

Reviewer #2: **Yes:**   Alexander Bello

# Editor report: Acceptance letter

**DOI:** 10.1371/journal.pntd.0014438.r005

**Authors:** David Safronetz

Dear Dr Lamula,

We are delighted to inform you that your manuscript, "Epidemiology, seroprevalence, and circulation of Chikungunya virus in Southern Africa (SADC region): A systematic review," has been formally accepted for publication in PLOS Neglected Tropical Diseases.

We have now passed your article onto the PLOS Production Department who will complete the rest of the publication process. All authors will receive a confirmation email upon publication.

The corresponding author will soon be receiving a typeset proof for review, to ensure errors have not been introduced during production. Please review the PDF proof of your manuscript carefully, as this is the last chance to correct any scientific or type-setting errors. Please note that major changes, or those which affect the scientific understanding of the work, will likely cause delays to the publication date of your manuscript. Note: Proofs for Front Matter articles (Editorial, Viewpoint, Symposium, Review, etc...) are generated on a different schedule and may not be made available as quickly.

Soon after your final files are uploaded, the early version of your manuscript will be published online unless you opted out of this process. The date of the early version will be your article's publication date. The final article will be published to the same URL, and all versions of the paper will be accessible to readers.

For Research Articles, you will receive an invoice from PLOS for your publication fee after your manuscript has reached the completed accept phase. If you receive an email requesting payment before acceptance or for any other service, this may be a phishing scheme. Learn how to identify phishing emails and protect your accounts at https://explore.plos.org/phishing.

Thank you again for supporting open-access publishing; we are looking forward to publishing your work in PLOS Neglected Tropical Diseases.

Best regards,

Shaden Kamhawi

co-Editor-in-Chief

PLOS Neglected Tropical Diseases

Paul Brindley

co-Editor-in-Chief

PLOS Neglected Tropical Diseases
